# Adsorption and Dynamic Characteristics of PFAS Mixtures with Kaolinite: Molecular Insights into the Impact of Chain Length and Functional Group

**DOI:** 10.1021/acs.est.5c01046

**Published:** 2025-07-10

**Authors:** Narasimhan Loganathan, Christina E. Schumm, Mary K. O’Reilly, Angela K. Wilson

**Affiliations:** † Department of Chemistry and the MSU Center for PFAS Research, 3078 Michigan State University, East Lansing, Michigan 48824, United States; ‡ Department of Physical Sciences, Truman State University, Kirksville, Missouri 63501, United States

**Keywords:** PFOS, PFOA, PFH*x*S, PFAS mixtures, Soils, Silicates, Kaolinite, Molecular Dynamics

## Abstract

The widespread distribution of Per-fluoroalkyl and polyfluoroalkyl substances (PFAS) in soil and sediments is a major concern for environmental and human health. Though significant progress has been made to understand the behavior of PFAS in near- and subsurface regions, much of the current insight is limited to homogeneous solutions of PFAS. However, PFAS often exists within PFAS mixtures in soil environments, and there is far less knowledge about these mixtures. In this work, the impact of binary mixture compositions of PFAS on the interfacial adsorption, structure, and diffusion characteristics with the soil mineral kaolinite has been investigated. For the first time, the effects of terminal functionalities and PFAS concentrations in a binary mixture on surface complexation have been examined on the molecular level. Importantly, the diffusion behavior of carboxylate PFAS molecules in a surface adsorbed cluster is dependent on the number of sulfonated PFAS molecules within the cluster.

## Graphical abstract

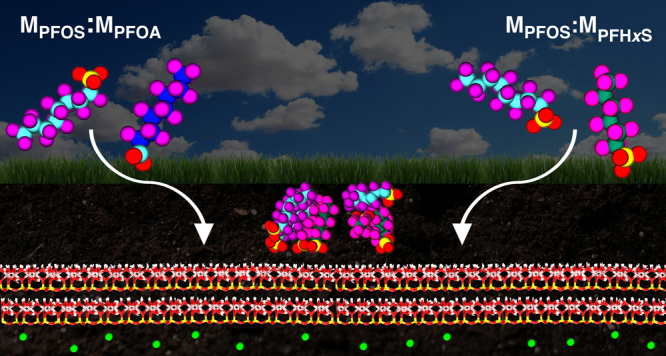

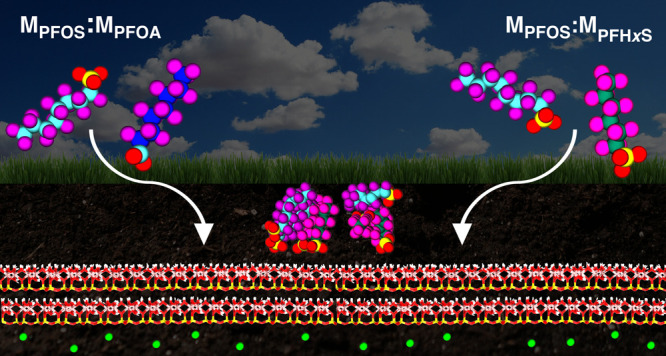

## Introduction

The widespread use of per- and polyfluoroalkyl substances (PFAS) in industrial, commercial, and domestic applications for over six decades has raised major concerns for environmental, human, and animal health.^1–8^ The extreme stability of PFAS molecules toward degradation techniques including thermal, photolytic, biological, and chemical approaches stems from the unique PFAS structure, encompassing both strong C–F bonds and terminal functionalities.^2^ Consequently, PFAS molecules have been identified in environmental matrices including soils, sediments, ground- and surface waters, and the atmosphere—all of which represent exposure pathways for humans.^9–15^ Toxicological studies have linked the exposure of PFAS with detrimental health impacts including cancer, reduced kidney function; cardiovascular diseases; and thyroid, developmental, and reproductive disorders.^4,16–21^ As a result, significant attention is being directed toward regulating PFAS usage globally. For instance, the U.S. Environmental Protection Agency (EPA) has banned the use of perfluorooctanesulfonic acid (PFOS) and perfluorooctanoic acid (PFOA) and has suggested regulations on the concentration of short and new-age PFAS alternatives in water sources.^22^

Although significant progress has been made toward developing adsorbent materials for removing PFAS from water sources,^23–27^ mitigation and remediation strategies for PFAS in contaminated soils are quite limited. This limitation may be attributed to the complex nature of soil constituents. Much of the knowledge into the behavior of PFAS in soils was obtained via distribution coefficients determined through batch adsorption studies.^10,28–35^ For instance, Milinovic et al.^31^ examined the adsorption of PFOS, PFOA, and perfluorobutanesulfonic acid (PFBS) in six different soil samples and found preferential adsorption of long-chain PFAS molecules over short-chain ones, irrespective of soil characteristics. A recent study by Li et al.^36^ showed that the adsorption of PFOS is higher for soil samples with significant clay fractions. Furthermore, Fabregat-Palau et al.^32^ developed a sorption model to account for the significant role of soil mineral phases in adsorbing PFAS to overcome the underestimation of distribution coefficients for PFAS in soils with significant mineral fractions.

For PFAS sorption with minerals, Hellsing et al.^33^ suggested that the adsorption of PFAS with alumina minerals is substantially higher than with a silica surface. On the other hand, studies by Zhao et al.^29^ demonstrated that sulfonate PFAS molecules exhibit stronger sorption properties than carboxylate PFAS in smectites and kaolinite. Similarly, Zhang et al.^28^ showed that kaolinite exhibits a higher adsorption capacity for PFOS than montmorillonite. In recent years, studies have provided insight into the adsorption, energetics, and diffusion of PFAS molecules at the surfaces of soil minerals using molecular dynamics (MD) simulations.^37–40^ Our previous MD studies indicated the formation of large, aggregated clusters for long-chain PFAS (C > 7) and new age PFAS alternatives (GenX and fluorotelomer sulfonates) at the surfaces of kaolinite that are stable for longer periods of time.^37–39^ Prior studies have shown that hydrophobic interactions drive the adsorption of PFAS (legacy and new age) at the surfaces of montmorillonite.^38–40^ Though these studies have contributed toward progress in understanding the behavior of PFAS at mineral surfaces, all of these studies considered PFAS in a single-phase composition.

At any contaminated site, it is likely that PFAS molecules exist in mixture compositions, where multiple PFAS with varying chain length and terminal functionalities are present in different concentrations. Thus, in any mitigation approach, it is important to consider mixtures. For example, in recent years, the removal of PFAS mixtures from drinking water using a variety of adsorbent materials has been pursued.^25,41–46^ Ilango et al.^44^ reported the use of modified granular activated carbon to remove PFAS mixtures, providing a more effective approach than activated carbon.

Despite these steps toward mitigation, insight into the impact of PFAS mixtures on their adsorption at soil mineral interfaces is limited. Moreover, these studies have been focused on understanding the influence of PFAS mixtures on the adsorption and transport properties of PFAS in bulk soil samples.^47–52^ For instance, Huang et al.^49^ suggest that the adsorption of PFOS in the solid phase is not influenced by the presence of other PFAS molecules. Similarly, Liao et al.^53^ showed that the distributions of PFOS and PFNA in solid phases are not significantly different in single-phase and binary mixtures. In contrast, a recent study by Garza-Rubalcava et al.^48^ indicated that the retention characteristics of PFOA in variably saturated pores decreased in the presence of PFOS due to the competition for the pore sites in solids. Similarly, Umeh et al.^47^ suggested that the high affinity of PFOS in water-saturated soils could substantially limit the interaction of PFOA and perfluorohexanesulfonic acid (PFH*x*S) with soils in mixtures and is prominent in highly concentrated mixtures. ***The widely varying outcomes from these studies indicate the need for a molecular level understanding of the adsorption and diffusion properties of PFAS mixtures at different concentrations in soils, especially with soil minerals, which dictate their transport properties in near- and subsurface regions***.

Herein, one of the first investigations to address the competitive adsorption behavior of PFAS molecules is provided using molecular dynamics simulations. Three PFAS that vary in chain length and functionalities, namely PFOS, PFOA, and PFH*x*S, have been considered in the mesopores of kaolinite, an important soil mineral that is identified in regions where PFAS contamination has been reported, using MD simulations. The main objective of the study is to critically evaluate factors that impact the adsorption and diffusion characteristics of PFAS in mixtures and how the sorption properties vary with concentrations of PFAS. These insights are vital toward the determination of the distribution of PFAS in near- and subsurface regions and how the distribution varies in mixtures.

## Simulation Methods

Kaolinite is a 1:1 layered aluminosilicate mineral, where a layer of Si tetrahedra (T) is coordinated to a layer of Al octahedra (O). The kaolinite structure examined in this study has a structural composition of Al_4_Si_4_O_10_(OH)_8_ and is based on the optimized structure from a study by Cygan et al.^54^ Kaolinite, a neutral mineral, exhibits both hydrophilic and hydrophobic surface characteristics. This is primarily due to the presence of hydroxyl groups at the “O” side resulting in hydrophilic character and the lack of isomorphic substitution of basal structural atoms along the “T” side, providing the surface with a hydrophobic character. The external surfaces of kaolinite were built by cleaving the structure along the basal (001) plane, resulting in interactions of the hydrophobic and hydrophilic surfaces of kaolinite with the solution phase.

The simulation cell included two kaolinite layers with a total thickness of ∼1.4 nm and a lateral dimension of 46.08 nm^2^. The distance between the cleaved surfaces was ∼13.6 nm. A large nanopore separation between the kaolinite surfaces eliminates interaction between the layers when periodic boundary conditions are applied. The basal (001) surface was saturated with water molecules to replicate bulk-like solution environments (1.0 g/cm^3^). Kaolinite models for simulations are described in prior studies.^37,38^ Similar large-scale pore models of kaolinite have been utilized previously to address the adsorption and diffusion of alkali and alkaline earth metal ions and organic species, demonstrating the utility of such models.^55–59^

The PFAS molecules considered in this study, PFOA, PFOS, and PFH*x*S, represent both long- and short-chain types. To understand the influence of chain length and functional group on the interfacial adsorption and dynamical properties of PFAS, two types of binary mixtures were examined: (i) PFOS/PFOA and (ii) PFOS/PFH*x*S. For each type of binary mixture, simulation models with five different concentration ratios were constructed corresponding to 87.5/12.5 (%), 75/25 (%), 50/50 (%), 25/75 (%), and 12.5/87.5 (%). Since the realistic concentration of PFAS mixtures in soils is not known to our knowledge, the current study examined the whole range of mixture concentrations. The interfacial properties of single-phase PFAS molecules at the kaolinite surface were reported earlier.^37^ Irrespective of the binary mixture concentration ratio, 16 PFAS molecules were included in the nanopore region. All PFAS molecules were distributed in four distinct regions of the nanopore, separated by distances of 2–3 nm. Each region contains four PFAS molecules separated by several water layers. The different PFAS molecule types were distributed in each mixture uniformly throughout the bulk solution region. All PFAS molecules were modeled in a deprotonated state to simulate near-neutral pH conditions, and the resulting net-negative charges were offset by the incorporation of an equivalent number of Ca^2+^ ions. A schematic representation of the simulation model for the equimolar mixture of PFOS/PFH*x*S, PFAS molecules examined, and the structural details of kaolinite are provided in the Supporting Information (Figure S1a–c).

MD simulations were performed at 300 K and 1 bar using the LAMMPS simulation package.^60^ For each binary system, the simulations were initially conducted in the *NPT* ensemble [number of atoms (*N*), pressure (*P*,) and temperature (*T*) constant] and subsequently in the *NVT* ensemble [number of atoms (*N*), volume (*V*), and temperature (*T*) constant]. During the simulation run, the temperature and pressure were controlled using the Nose-Hoover thermostat and barostat.^61,62^ Three-dimensional periodic boundary conditions were employed. A cutoff distance of 10 Å was employed to compute short-range nonelectrostatic interactions, and the particle–particle–particle mesh (PPPM) summation method was used to calculate long-range electrostatics with an accuracy of 1e^–6^.^63^ The interatomic interactions of kaolinite and metal ions were computed using the CLAYFF force field (including Al–O–H bending potential) and the PFAS molecules were modeled using an AMBER interaction potential.^64–67^ SPC interaction potential was used to represent the water molecules.^68^ These interaction potentials were chosen based on their demonstrated utility in providing consistent results with experimental data for clay minerals.^59,69–71^

Irrespective of the binary mixture composition, all kaolinite models were simulated for 55 ns with a time step of 1 fs. Initially, a 15 ns (10 ns equilibration and 5 ns data production) simulation was carried out in the *NPT* ensemble to obtain the equilibrium cell dimension corresponding to the bulk water density. Subsequently, *NVT* simulations were performed for another 40 ns, which includes 10 ns for equilibration and 30 ns for data production. The data from the last 5 ns of the *NVT* run were collected at every 10 fs to determine the complexation structure and dynamics of all of the species in the interfacial region.

## Results and Discussion

### Atomic Density Profiles (ADPs)

The ADPs of PFOS/PFOA (Figures 1a–f and S2a–d) and PFOS/PFH*x*S (Figures 2a–f and S3a–d) binary mixtures as functions of distance normal to the basal surface of kaolinite demonstrate that the PFAS molecules are exclusively adsorbed at the hydroxyl layer of kaolinite, irrespective of the nature of functional group and concentrations. The charge-compensating Ca^2+^ ions are adsorbed near the siloxane surface. The surface adsorption of deprotonated sulfonate (PFOS/PFH*x*S) and carboxylate groups (PFOA) is dominated by the H-bonding interactions with the surface hydroxyl groups and are consistent with previous studies that reported the adsorption of polar organic molecules near the hydrophilic side of kaolinite, in the absence of salts.^59^ However, near-surface distributions of ADPs vary substantially between the mixtures (PFOS/PFOA and PFOS/PFH*x*S) and are discussed separately (Figures S4 and S5). The ADPs of Ca^2+^ ions and H_2_O molecules are discussed in the Supporting Information (Figure S6).

**Figure 1:**
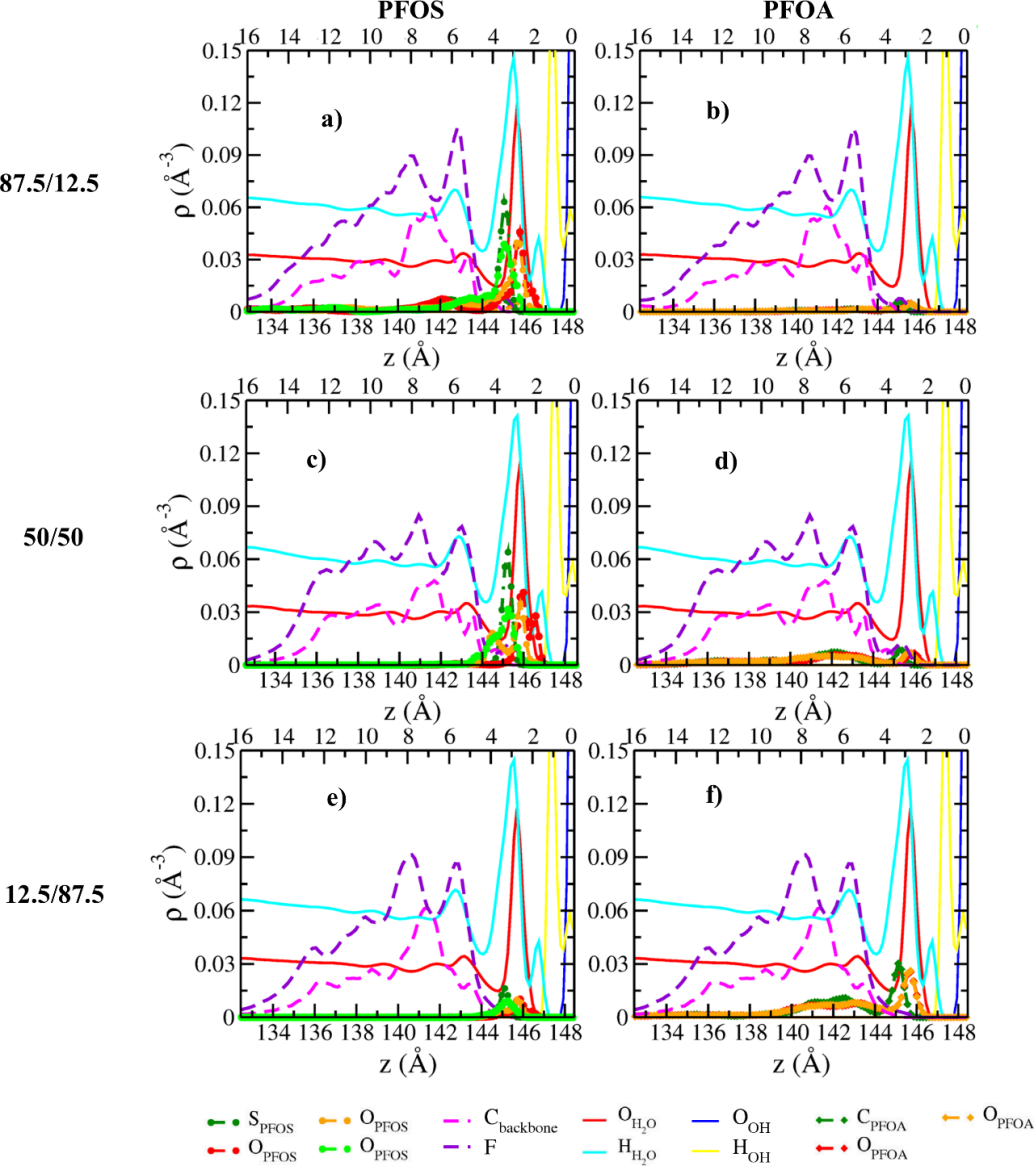
Computed ADPs of PFOS, PFOA, and H_2_O molecules in Ca-kaolinite as functions of distance normal to the basal hydroxyl surface for three different PFOS–PFOA binary mixture compositions. (a and b) 87.5/12.5 (%); (c and d) 50/50 (%); (e and f) 12.5/87.5 (%). Left and right columns represent PFOS and PFOA at different concentrations, respectively. Dashed lines represent the carbon backbone (pink) and fluorine (purple) of the PFAS molecule. The “*z*” values in the bottom *x*-axis represent the actual positions of the PFAS and H_2_O molecules in the simulation box. The *z* values in the top *x*-axis correspond to the distances of PFAS and H_2_O molecules from the plane corresponding to the peak maxima of the basal surface hydroxyl oxygen (O_OH_) atoms of kaolinite which is set as the origin on the right side (0.0 Å).

**Figure 2:**
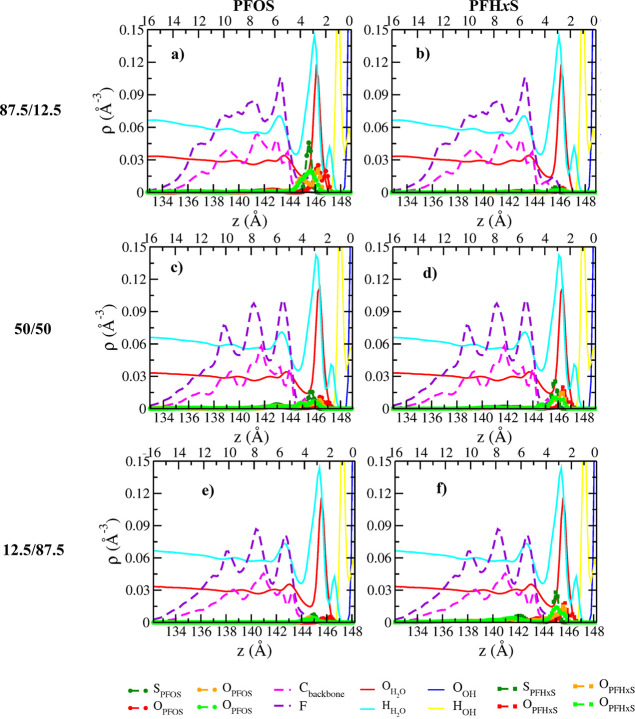
ADPs of PFOS, PFH*x*S, and H_2_O in Ca-kaolinite as functions of distance normal to the basal hydroxyl surface for three different PFOS/PFH*x*S binary mixture compositions. (a and b) 87.5/12.5 (%); (c and d) 50/50 (%); (e and f) 12.5/87.5 (%). Left and right columns represent PFOS and PFH*x*S at different concentrations, respectively. Dashed lines represent the carbon backbone (pink) and fluorine (purple) of PFAS. The “*z*” values in the bottom *x*-axis represent the actual positions of the PFAS and H_2_O molecules in the simulation box. The *z* values in the top *x*-axis correspond to the distances of PFAS and H_2_O molecules from the plane corresponding to the peak maxima of the basal surface hydroxyl oxygen (O_OH_) atoms of kaolinite which is set as the origin on the right side (0.0 Å).

#### PFOS/PFOA

ADPs of PFOS (Figures 1a–1f, S2a–d, and S4a–o) are characterized by a dominant peak at ∼2.7 Å and a minor peak at ∼2.0 Å from the basal hydroxyl surface, for all concentrations in the mixture. For the equimolar concentrations of PFOS and PFOA, the peak at ∼2.0 Å is well-defined. Both peaks exhibit surface coordination. Similar intensity peaks at ∼2.7 Å for two O_SO_3__ atoms demonstrate that most PFOS molecules are coordinated to the surface by two distinct H-bonding interactions (orange circle in Figure S7). Due to the flexible nature of the hydroxyl groups, a small fraction of PFOS molecules have one O_SO_3__ atom located at the center of the hydroxyl cavities exhibiting H-bonding interaction resulting in the minor peak closer to the surface at all concentrations (blue circle in Figure S7). The third of the O_SO_3__ atoms are predominantly located away from the surface with peaks only at z > 3.3 Å and hence do not participate in surface coordination (Figure S4). The exception in the ADPs is the presence of a shoulder peak (*z* = 2.7 Å) for the third O_SO_3__ (green) atoms in the 75/25 binary mixture (Figure S4e) which could be attributed to (i) a fraction of PFOS molecules being coordinated to the surface with all three O_SO_3__ atoms; (ii) among the surface coordinated PFOS molecules through two O_SO_3__ atoms, a fraction of the molecules are coordinated with the surface through O_SO_3__ (red and green). Based on the decreased intensity of one of the O_SO_3__ (orange) atoms and the presence of a peak for O_SO_3__ (green), as shown in Figures S2a and S4e, it is highly probable for scenario (ii) to be more favorable than (i). The largely similar orientation of the SO_3_^–^ groups (∼18°, Figure S8a) and the absence of peaks near 0° with respect to the surface normal for 75% PFOS and 25% PFOA systems with other mixture compositions indicates that the probability of PFOS having all three O_SO_3__ atoms coordinated to the surface is not feasible.

The PFOA adsorption is dominated by interactions between both the O_COO_ atoms and the basal hydroxyl surface, which is evident by the presence of similar intensity peaks at ∼2.8 Å from the surface, irrespective of their concentration in the mixtures (Figures 1a–f and S4). Furthermore, the computed orientations of surface adsorbed COO^–^ groups with respect to surface normal suggest that PFOA molecules prefer near-perpendicular orientations (∼19°, Figure S8b) for most concentrations. The broad orientational distribution of COO^–^ groups implies that the surface adsorbed molecules exhibit constant rocking motions toward and away from the basal hydroxyl surface. For the lowest concentrations (87.5/12.5) of PFOA, PFOA molecules favor two different orientations (∼12° and ∼55°) with respect to the surface normal, indicating that the surface adsorbed molecules experience larger librational motion. The ADP peak intensities of surface adsorbed C_COO_ and O_COO_ atoms of PFOA increase only at higher concentrations (>50%), demonstrating that the surface adsorption is dominated only by PFOS until equimolar concentrations (Figures S4 and S5) are reached. The reported interfacial structure for PFOA is consistent with our study on single-phase PFOA studies on kaolinite.^37^

#### PFOS/PFH*x*S

Irrespective of concentration examined, the ADPs of PFOS and PFH*x*S are characterized by distinct peaks at ∼2.0 and 2.7 Å from the basal hydroxyl surface (Figures 2a–f, S3a–d, and S5a–o). Unlike the PFOS/PFOA mixture, the peak at ∼2.0 Å is prominent at all concentrations, indicating that sulfonated PFAS have one of their O_SO_3__ atoms at the center of hydroxyl cavities (Figure S7), irrespective of chain length, consistent with previous studies with PFOS at kaolinite surfaces.^37^ Similarly, PFOS and PFH*x*S at ∼2.7 Å have two of their O_SO_3__ atoms coordinated to the surface directly on top of the hydroxyl group (Figure S7). Consequently, the orientations of PFOS and PFH*x*S with respect to the surface normal are similar (Figure S8c,d). However, the third O_SO_3__ (green) atoms of PFOS are located predominantly at distances >3.2 Å sharing the same plane of S_SO_3__ and do not have any interactions with the surface. In contrast, the third O_SO_3__ atoms (green) of PFH*x*S exhibit peaks at ∼2.8 Å and at >3.2 Å for all concentrations (Figure S5). The former peak suggests that all three O_SO_3__ probe the surface but the PFH*x*S molecules predominantly are coordinated only with their two O_SO_3__ atoms during the simulation. In addition, the lack of peaks near 0° in the orientations of PFH*x*S indicate that there are no molecules with all three O_SO_3__ atoms coordinated to the surface at all concentrations (Figure S8d). Unlike in PFOS/PFOA mixtures, the intensity of PFOS decreases and that of PFH*x*S increases with an increase in the concentration of PFH*x*S in the mixture.

### Planar Density Map (PDM)

PDMs of S_SO_3__, C_COO_, and H_2_O in PFOS/PFOA (Figures 3a–f and S9–S13) and PDMs of S_SO_3__ and H_2_O in PFOS/PFH*x*S (Figures 4a–f and S14–S18) mixtures demonstrate that PFAS molecules are located at distinct regions on the basal hydroxyl surface. The degree of localization strongly depends on the concentration and terminal functionalities of the mixture. The strong localized adsorption characteristics for both types of mixtures indicate the formation of large-aggregated clusters that are stable for long periods of time, especially for systems with higher concentration of PFOS. Consequently, PDMs of H_2_O show vacant regions on the hydroxyl surface, coinciding well with the location of PFAS molecules. However, the nature of aggregated clusters varies with the concentration and mixture composition.

**Figure 3:**
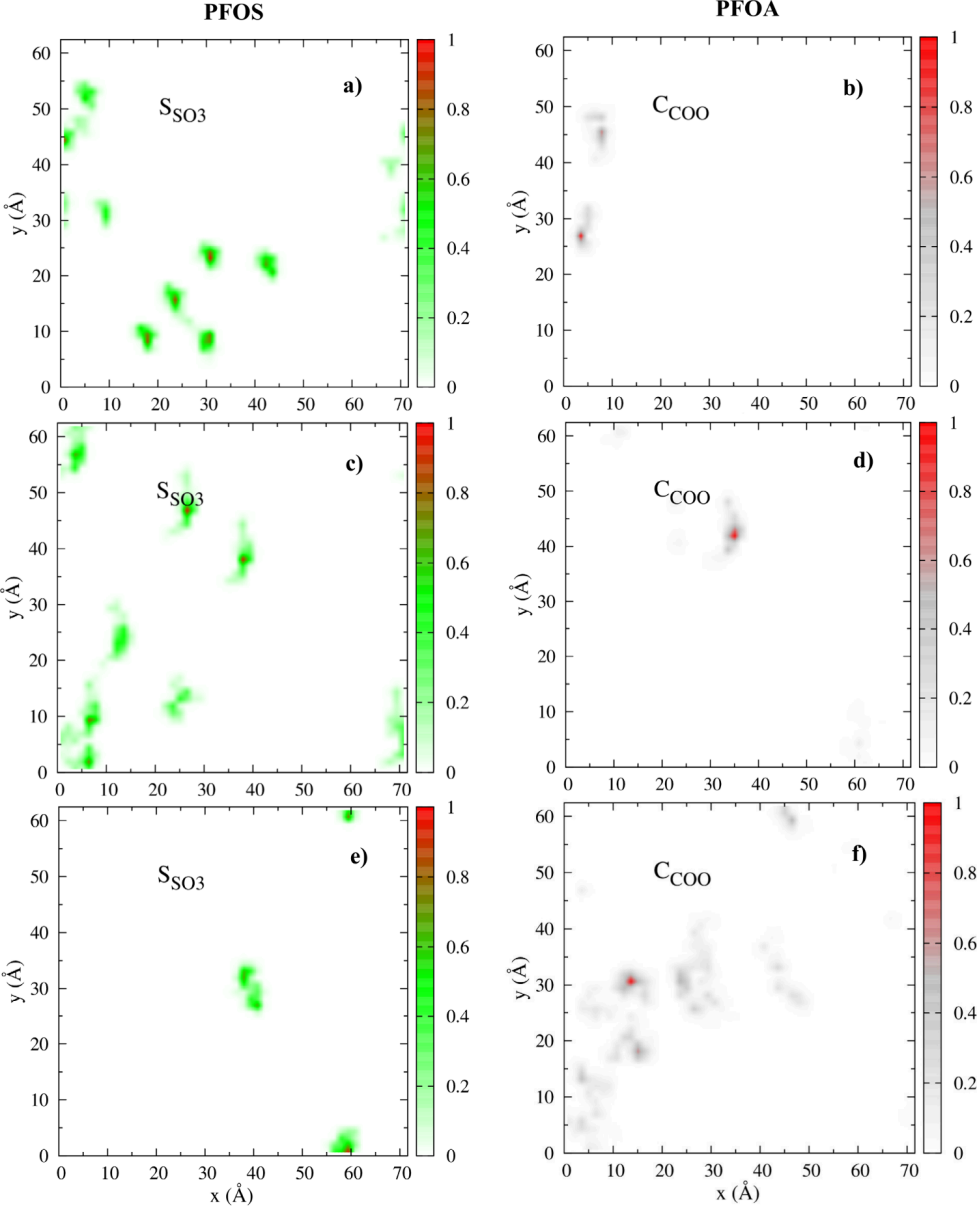
PDMs of surface adsorbed PFOS and PFOA in Ca-kaolinite on the basal hydroxyl surface at different PFOS- PFOA composition for (a and b) 87.5/12.5 (%), (c and d) 50/50 (%), and (e and f) 12.5/87.5 (%). Left column: S_SO_3__ of PFOS; right column: C_COO_ of PFOA.

**Figure 4:**
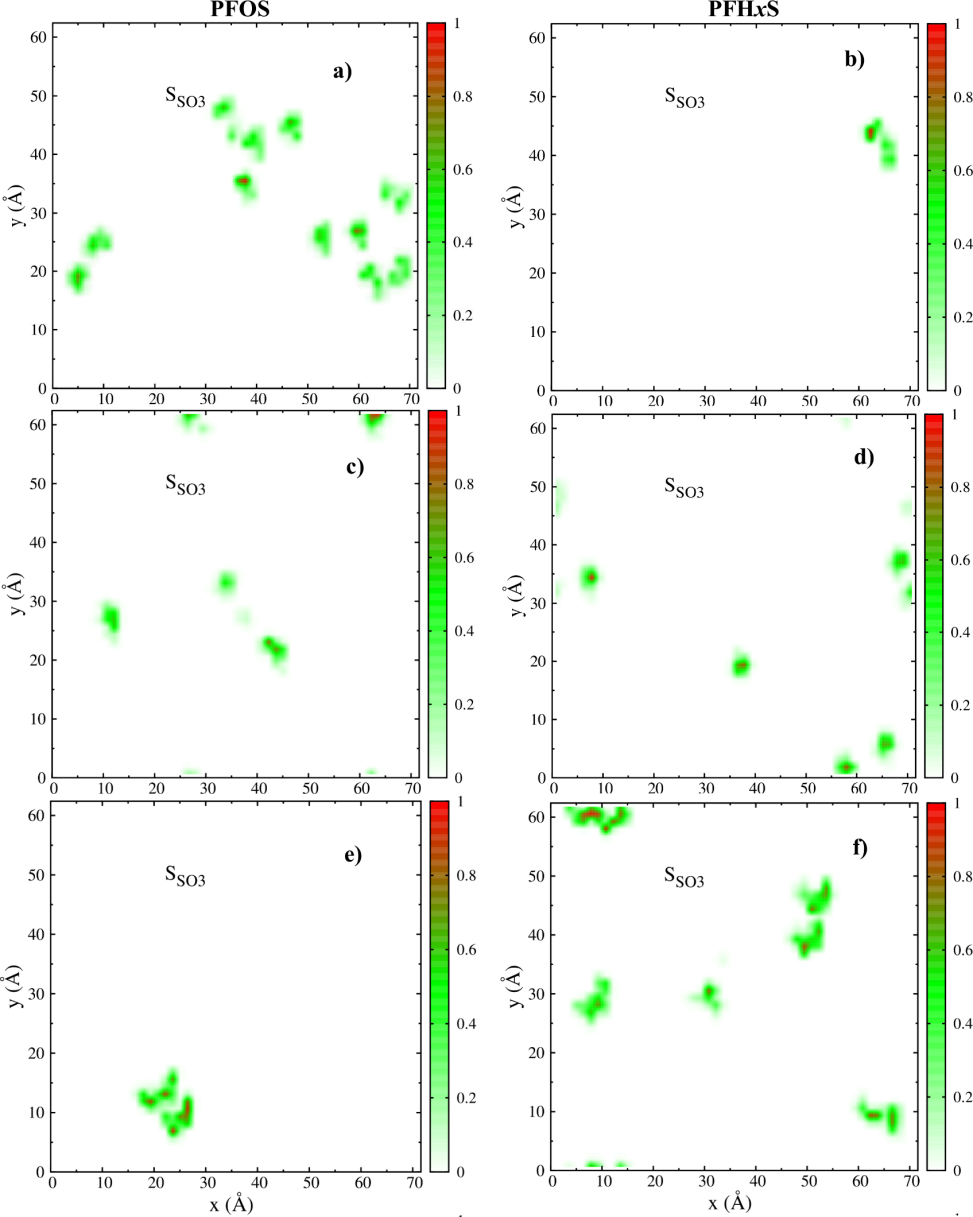
PDMs of surface adsorbed PFOS and PFH*x*S in Ca-kaolinite on the basal hydroxyl surface at different PFOS-PFH*x*S composition for (a and b) 87.5/12.5 (%); (c and d) 50/50 (%); (e and f) 12.5/87.5 (%). Left column: S_SO_3__ of PFOS; right column: S_SO_3__ of PFH*x*S.

#### 87.5/12.5%

PDMs of S_SO_3__ show that PFOS molecules (Figure 3a) in PFOS/PFOA mixtures are located in two distinct regions of the hydroxyl surface, and the strong PFOA density for C_COO_ in Figure 3b indicates that those molecules are adsorbed in clusters with PFOS and also exhibit H-bonding interaction with the basal hydroxyl surface. The corresponding O_SO_3__ and O_COO_ are shown in Figure S9a–e. From Figure S9h, the interfacial adsorption environment is characterized by two large clusters, nonamer (5) and heptamer (4) where the values in parentheses represent the average number of molecules showing direct coordination to the surface from each cluster, corroborating with the high-density regions of PDMs for S_SO_3__ and C_COO_. The nonamer is composed of PFOS while the heptamer has PFOS and PFOA. The nonsurface bound PFAS molecules in the clusters are adsorbed through hydrophobic interactions with the surface-bound PFAS (Figure S9g). Further details of how many PFOS and PFOA molecules exhibit direct coordination with the surface from each cluster are given in Table 1.

**Table 1:**
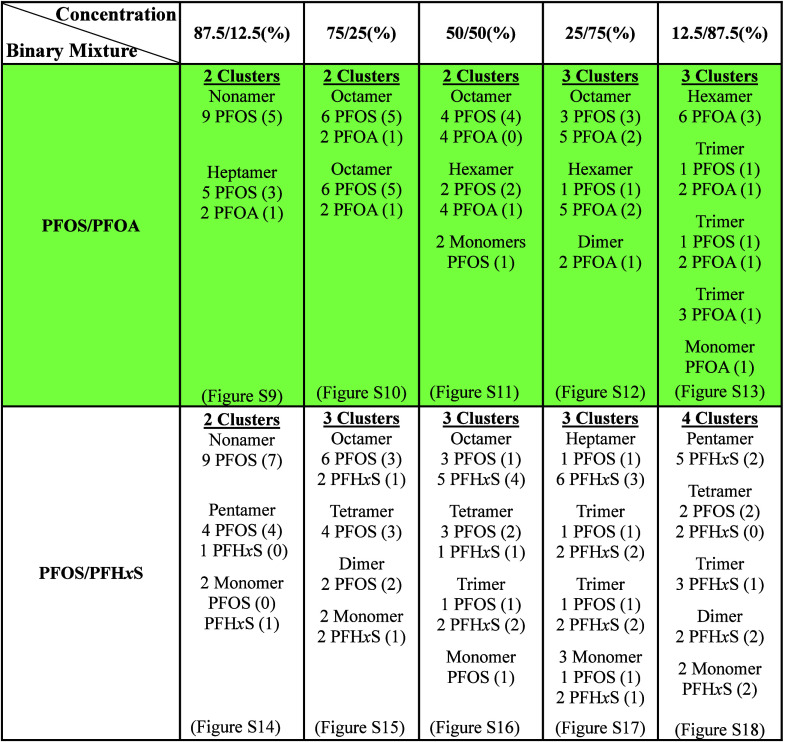
Stable Clusters Formed in PFOS/PFOA and the PFOS/PFH*x*S Binary Mixture at Different Concentrations

PDMs of S_SO_3__ in PFOS/PFH*x*S mixtures show predominantly two regions at the hydroxyl surface for the adsorption of PFAS molecules (Figure 4a,b). The strong density of S_SO_3__ for PFH*x*S in Figure 4b demonstrates surface coordination, but their location on the basal surface is significantly away from the dominant cluster regions. The adsorption environment in Figure S14i shows the formation of a nonamer (7), a pentamer (4), and 2 monomers that are stable for longer periods of time. The average coordination with the surface for each cluster is indicated in parentheses and is consistent with the reported surface-bound PDMs (Figure 4a,b). The remaining PFAS molecules are adsorbed with the surface adsorbed PFAS through hydrophobic interactions (Figure S14h). Notably, the nonamer is composed of only PFOS molecules (similar to PFOS/PFOA mixture), while the pentamer has PFOS and PFH*x*S (Table 1). The surface adsorbed PFH*x*S monomer is responsible for their isolated PDMs in Figure 4b. The corresponding PDMs O_SO_3__ of PFOS and PFH*x*S is shown in Figure S14a–f, indicating basal surface coordination.

#### 75/25%

The location of PDMs of S_SO_3__ and C_COO_ (PFOS/PFOA mixtures) in Figure S10a–j indicates that there are two clusters at the basal surface and each cluster is composed of PFOS and PFOA. The interfacial adsorption structure provides validation of our inference from PDMs with the presence of two octamers at the hydroxyl surface (Figure S10j). Each octamer cluster is made up of six PFOS molecules and two PFOA molecules, and the values in parentheses (Table 1) represent the average number of molecules from each cluster that exhibit direct coordination with the surface while the others are coordinated to the clusters through hydrophobic interactions (Figure S10i).

In contrast, although the PDMs of S_SO_3__ in PFOS/PFH*x*S are highly localized, their locations are not well correlated as in PFOS/PFOA which indicates the existence of additional clusters, especially for PFH*x*S (Figure S15a,e). In addition, the PDMs of H_2_O in Figure S15i show more vacant regions at the hydroxyl surface of kaolinite than in PFOS/PFOA (Figure S10h). Furthermore, the surface adsorbed clusters include an octamer (4), a tetramer (3), a dimer (2), and 2 monomers (1) (Figure S15k). The values in parentheses are consistent with the reported S_SO_3__ PDMs (Figure S15a,e) of PFOS and PFH*x*S molecules. The octamer is composed of 6 PFOS and 2 PFH*x*S molecules, while the tetramer and dimer are only made of PFOS. Both monomers are PFH*x*S.

#### 50/50%

The PDMs of S_SO_3_,_ C_COO_, and H_2_O for the equimolar mixtures of PFOS/PFOA illustrate a stable localized structure for the surface adsorbed clusters (Figures 3c,d and S11a–h). The interfacial adsorption structure shows the formation of an octamer (4), a hexamer (3), and two monomers (1) (Figure S11h). The octamer is composed of four PFOS and four PFOA molecules, while the hexamer consists of two PFOS and four PFOA. Both monomers of PFOS exhibit direct coordination with the surface. Table 1 lists the coordination contribution from each type of PFAS molecule in each cluster, which agrees well with the reported PDMs.

The PDMs of S_SO_3__, O_SO_3__, and H_2_O in PFOS/PFH*x*S indicate densities at different regions of the hydroxyl surface which could be attributed to a higher number of clusters than in PFOS/PFOA (Figures 4c,d and S16a–i). The surface adsorption structure indicates the presence of an octamer (5), tetramer (3), trimer (3), and monomer (1), while the nonsurface adsorbed molecules are part of the cluster through hydrophobic interactions (Figure S16h). The clusters have the following combination: (i) an octamer composed of three PFOS and five PFH*x*S; (ii) a tetramer of three PFOS and one PFH*x*S; and (iii) a trimer of one PFOS and two PFH*x*S (Figure S16i). The monomeric PFOS is surface bound.

#### 25/75%

From Figure S12, a larger fraction of PFOA molecules exhibit interaction with the hydroxyl surface of kaolinite, which could be attributed to their higher concentration in the PFOS/PFOA mixture. Although the concentration of PFOS is substantially low, PDMs of PFOS are highly localized (Figure S12a–d). In addition, Figure S12j illustrates that interfacial adsorption is characterized by an octamer (5), a hexamer (3), and one dimer (1). The octamer is composed of three PFOS and five PFOA, while the hexamer is composed of one PFOS and five PFOA. The dimer is composed only of PFOA. Despite the presence of five PFOA in the octamer and hexamer, only two exhibit direct coordination with the surface (Figure S12i), in contrast to all PFOS in those clusters which exhibit surface interaction (Table 1).

Although PDMs of PFOS show a localized interfacial adsorption structure, Figure S17a–d indicates a broad distribution for PFH*x*S across the basal hydroxyl surface. Strong PDMs of PFH*x*S result from their direct interaction with the hydroxyl surface (Figure S17e–h). The surface adsorbed clusters include a heptamer (4), two trimers (3), and three monomers (2) as shown in Figure S17k. The heptamer is composed of one PFOS and six PFH*x*S, while both trimers are comprised of one PFOS and two PFH*x*S.

#### 12.5/87.5%

PDMs of S_SO_3__ and O_SO_3__ in PFOS/PFOA mixtures are highly localized at the basal hydroxyl surface of kaolinite while PDMs of C_COO_ and O_COO_ are dispersed across the basal surface (Figures 3e,f and S13a–h). These locations demonstrate that PFOS prefers surface adsorption even at very low concentrations when compared to PFOA. The adsorption structure is composed of a hexamer (3), three trimers (1–2), and a monomer (Figure S13h). The hexamer is composed of only PFOA; however, not all of them exhibit direct coordination with the surface (Figure S13g). Instead, only half of them show direct interaction with the surface, while the others are coordinated through hydrophobic interactions (Table 1). Two of three trimers have one PFOS and two PFOA while the third is PFOA only. The adsorption structure corroborates well with PDMs for both PFAS.

PDMs of PFH*x*S are highly localized in the PFOS/PFH*x*S mixture and are located at distinct regions of the hydroxyl surface (Figures 4e,f and S18a–i). Additionally, PDMs indicate the formation of different clusters that vary in size based on the location. The adsorption environment shown in Figure S18i demonstrates the presence of five unique clusters including a pentamer (2), tetramer (2), trimer (1), dimer (2), and two monomers. The pentamer is composed of PFH*x*S molecules while the tetramer is comprised of two PFOS and two PFH*x*S. Other clusters consist of PFH*x*S. As for PFOS/PFOA, the PFOS prefer coordination with the basal hydroxyl surface of kaolinite even at low concentrations (Figure S18h).

### Diffusion Coefficients

Cluster size-dependent diffusion coefficients of PFAS were determined for the mixtures. Table 2 shows that the diffusion coefficients of large-aggregated clusters of PFAS are an order of magnitude smaller than those for H_2_O molecules, irrespective of mixture composition and terminal functionalities. However, the diffusion coefficients vary significantly for the mixtures. For example, the diffusion coefficient of clusters in PFOS/PFOA mixtures with ≥50% PFOS concentrations are an order of magnitude smaller than the diffusion coefficients for mixtures with <50% PFOS. These differences in diffusion coefficient magnitude could be attributed to the formation of large-aggregated clusters with high PFOS concentration in comparison to smaller aggregates at low PFOS concentration. For instance, at high concentrations of PFOS in the binary mixture, large-aggregates show a greater number of H-bonded interactions with the basal hydroxyl surface, thus resulting in restricted mobility. In contrast, the higher diffusion behavior of small, aggregated clusters stems from the limited number of direct H-bond interactions with the surface (Table 1).^37^

**Table 2:**
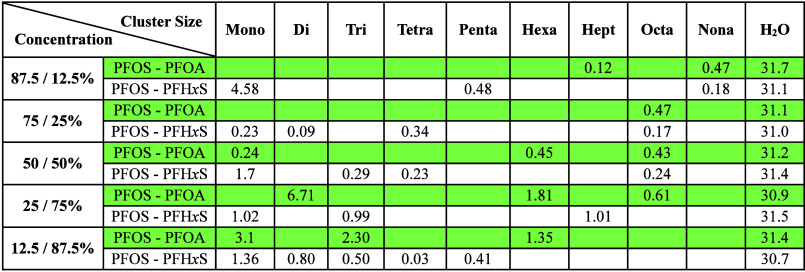
Diffusion Coefficients (10^–10^ m^2^/s) for Different Sized PFAS Clusters and H_2_O in Ca-Kaolinite at Five Concentrations^a^

a The errors associated with the diffusion values are within 1%.

Table 2 shows that irrespective of cluster size in PFOS/PFOA mixtures, the diffusion coefficients of clusters are similar until the mixture reaches equimolar composition, demonstrating that PFOS substantially hinders the mobility of the cluster. In addition, the diffusion coefficients for same-sized clusters increase with >50% PFOA in the mixture. For instance, the diffusion coefficients of hexamer in an equimolar mixture of PFOS/PFOA are ∼4 times smaller than those of the hexamer with 25% PFOS and 75% PFOA, despite having the same number of H-bond interactions with the surface. Such substantial differences could be associated with the higher number of surface bound PFOS interactions in the equimolar mixtures than at higher concentration of PFOA in the mixture.

Irrespective of cluster size, the diffusion coefficients for PFOS/PFH*x*S mixtures are substantially smaller than those for PFOS/PFOA mixtures. In addition, at all concentrations, the diffusion coefficients for PFOS/PFOA mixtures are significantly smaller than PFOS/PFH*x*S mixtures even for the same sized clusters. The decreased diffusion behavior of same sized clusters in PFOS/PFH*x*S relative to PFOS/PFOA can be attributed to the ability of sulfonate groups in PFOS and PFH*x*S to show stable coordination with the surface when compared to carboxylate groups (Figure S19d). The only exception in the diffusion coefficient is with the monomers (4.58) of 87.5/12.5(%) PFOS/PFH*x*S. Notably, for systems with the same-sized clusters, only the mean diffusion coefficients are reported. For example, the diffusion coefficient of trimers in the 25/75(%) binary mixture of PFOS/PFH*x*S represents the mean diffusion of all trimers in the system. Consequently, the higher diffusion of monomers in the 87.5/12.5(%) mixture of PFOS/PFH*x*S can be attributed to the reported mean values which include surface adsorbed PFH*x*S and nonsurface bound PFOS molecules. Note that the diffusion of surface adsorbed PFH*x*S in 87.5 PFOS/12.5 PFH*x*S (%) is substantially small (1.69 × 10^–10^ m^2^/s).

In comparing the diffusion coefficients of surface adsorbed monomers, the diffusion coefficients of sulfonated PFAS (PFOS/PFH*x*S) are substantially smaller than for PFOA. For instance, the surface adsorbed monomeric PFH*x*S diffusion in 12.5/87.5(%) PFOS/PFH*x*S mixture is ∼2.5 times slower than PFOA monomers identified in corresponding PFOS/PFOA mixtures. Thus, the diffusion coefficients are strongly influenced by the functional groups. Notably, irrespective of the mixture compositions, the diffusion coefficients reported for PFOS in mixtures with >50% PFOS are very similar to the ones reported for single-phase PFOS studies with kaolinite.^37^

## Environmental Implications

Gaining comprehensive mechanistic insights into the effect of PFAS mixtures on their environmental distribution in near- and subsurface regions is essential to the development of effective remediation techniques. This study is one of the first investigations to unravel the interactions that dictate the preferential adsorption of PFAS from a mixture composition with kaolinite. In addition, the concentration-dependent interfacial adsorption and diffusion behavior of PFAS mixtures with varying chain length and terminal functionalities was reported for the first time using MD simulations. Irrespective of the binary mixture composition examined, deprotonated PFAS are exclusively adsorbed near the hydroxyl surface of kaolinite and are facilitated by H-bonding interactions. Importantly, the large aggregated clusters formed at the kaolinite surface can encompass two different types of PFAS molecules. However, the size of the clusters varies depending on the mixture composition and the initial thermodynamic conditions employed in the simulations. Irrespective of concentration, the number of aggregated clusters for binary mixtures having only sulfonates as terminal groups but varying chain length is higher than binary mixtures with same chain length but with different functionalities (SO_3_^–^/COO^–^). Moreover, the aggregated clusters are highly localized at the interfacial region particularly when the sulfonate composition is ≥50% in the binary mixture. Consequently, despite having higher number of clusters for binary mixtures with PFOS/PFH*x*S, their diffusion is substantially restricted in comparison to PFOS/PFOA mixture, which could be due to the ability of sulfonate groups in PFH*x*S molecules to exhibit direct interaction with the hydroxyl surface of kaolinite.

Thus, this study indicates that the interfacial structure of PFAS mixtures could vary substantially, depending on the concentration of individual PFAS in each composition. Furthermore, the mobility of PFAS in the interfacial region could not be correlated to the size of the clusters; instead, there is strong reliance on the type of functionalities in the binary mixture and total number of surface interactions that the PFAS molecules can exhibit with the surface. Since both H-bonding and hydrophobic interactions are the primary driver for the surface adsorption, it should be noted that their interfacial adsorption structure and diffusion characteristics could be substantially influenced by the presence of soil organic matter (SOM). The hydrophobic regions in SOM could either promote the adsorption of PFAS by being part of the surface adsorbed clusters or compete for the surface adsorption and limit the interaction of PFAS with the surface and should be examined.

## Supplementary Material



## Notes

Schematic representation of the initial state of the simulation cell; discussion of the ADPs of Ca^2+^ ions and H_2_O molecules; orientational distribution of surface adsorbed PFOS, PFOA, and PFH*x*S in kaolinite for different binary mixtures; PDMs of “O” atoms in PFOS, PFOA, and PFH*x*S at different concentrations (PDF)
