# Supplementary material for: Adsorption and Dynamic Characteristics of PFAS Mixtures with Kaolinite: Molecular Insights into the Impact of Chain Length and Functional Group
Source: Environ Sci Technol. 2025 Jul 10;59(28):14637–48. doi: 10.1021/acs.est.5c01046 (PMC12288066; doi:10.1021/acs.est.5c01046)
Supplement: Supplementary file 1 [file es5c01046_si_001.pdf]

Adsorption and Dynamic Characteristics of PFAS Mixtures with Kaolinite – Molecular Insights into the Impact of Chain Length and Functional Group

Narasimhan Loganathan[a], Christina E. Schumm[a], Mary K. O' Reilly[a,b] and Angela K. Wilson[a]*

[a)] Department of Chemistry and the MSU Center for PFAS Research, Michigan State University, East Lansing, Michigan 48824, United States

[b)] Department of Physical Sciences, Truman State University, Kirksville, Missouri 63501, United States

[*)] **Corresponding author's email:** akwilson@msu.edu

Summary: Pages – 23, Figures – 19, Tables - 1

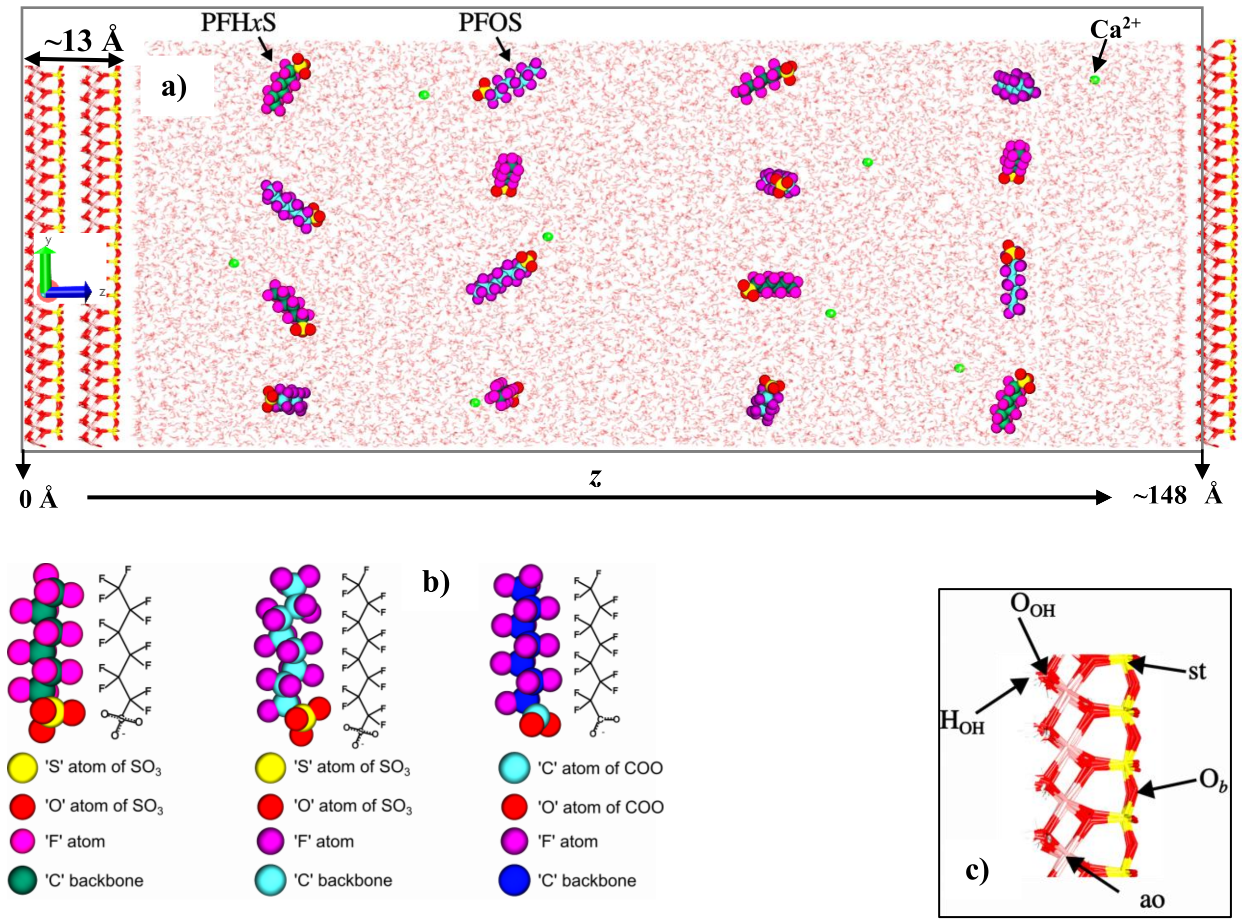

Figure S1: a) Schematic representation of the simulated equimolar (50/50) mixture of PFOS and PFH*x*S system at time *t*=0 ns. b) structure of PFH*x*S, PFOS and PFOA molecules. c) representation of different structural atoms of kaolinite. Color codes: pink sticks – Al octahedra; red sticks – hydroxyl and surface 'O' atoms; white sticks – hydroxyl 'H' atoms. Ca^{2+} ions are shown in light green spheres.

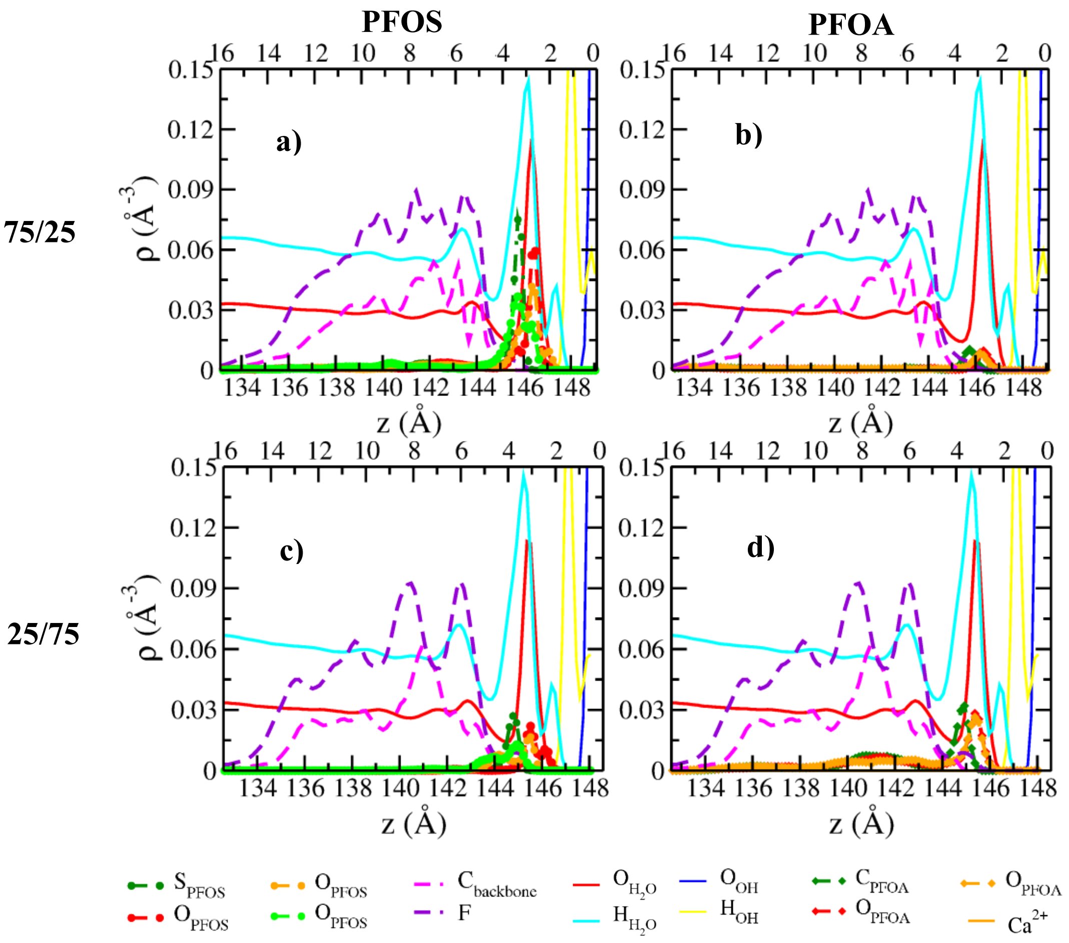

Figure S2: Computed ADP's of PFOS, PFOA and H_2O molecules in Ca-kaolinite as functions of distance normal to the basal hydroxyl surface for three different PFOS-PFOA binary mixture compositions. a) and b) - 75/25 (%); c) and d) – 25/75 (%).The '*z*' values in the bottom x-axis represents the actual positions of the PFAS and H_2O molecules in the simulation box (see Figure S1a). The '*z*' values in the top x-axis corresponds to the distances of PFAS and H_2O molecules from the plane corresponding to the peak maxima of the basal surface hydroxyl oxygen (O_{OH}) atoms of kaolinite which is set as the origin on the right side (0.0 Å).

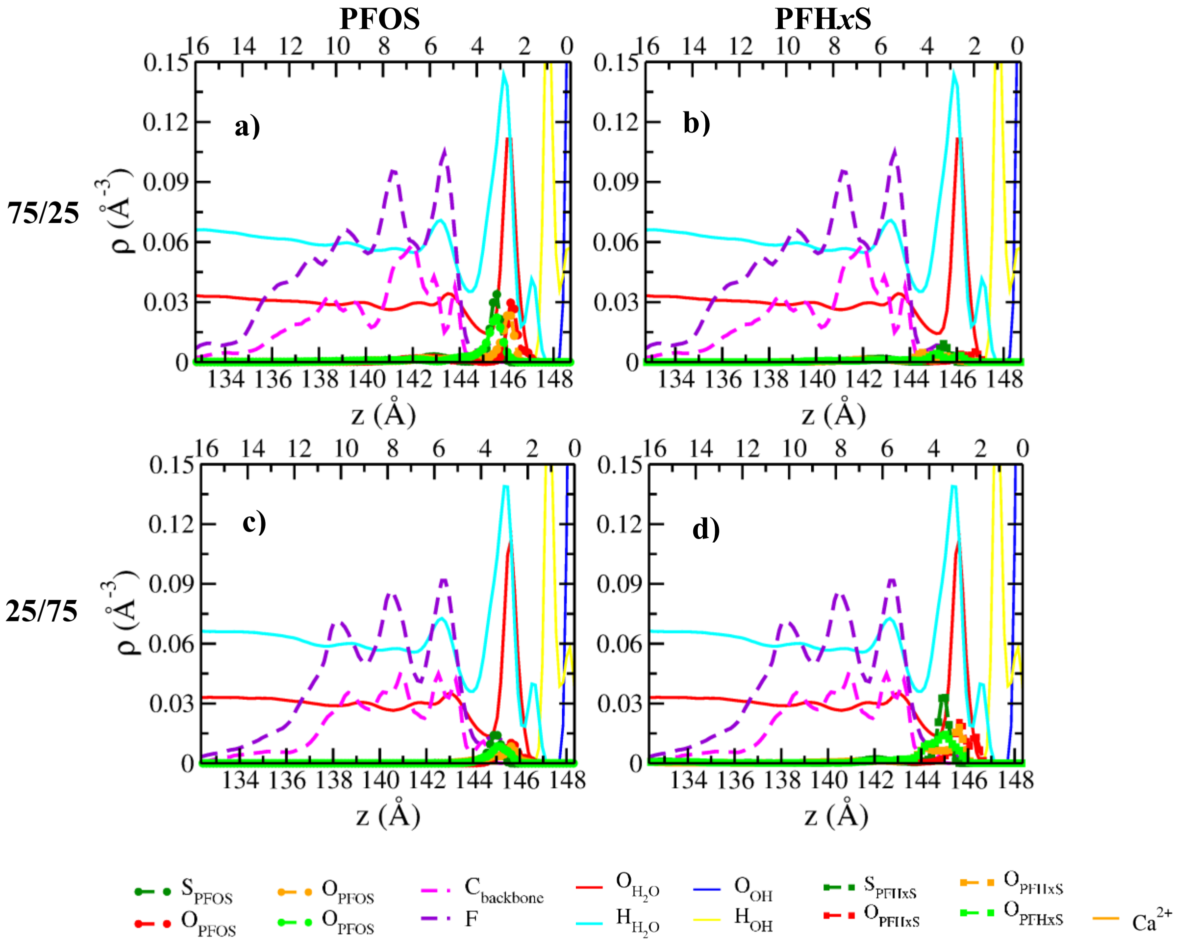

Figure S3: Computed ADP's of PFOS, PFH*x*S and H_2O molecules in Ca-kaolinite as functions of distance normal to the basal hydroxyl surface for three different PFOS- PFH*x*S binary mixture compositions. a) and b) – 75/25 (%); c) and d) – 25/75 (%). The '*z*' values in the bottom x-axis represent the actual positions of the PFAS and H_2O molecules in the simulation box (see Figure S1a). The '*z*' values in the top x-axis corresponds to the distances of PFAS and H_2O molecules from the plane corresponding to the peak maxima of the basal surface hydroxyl oxygen (O_{OH}) atoms of kaolinite which is set as the origin on the right side (0.0 Å).

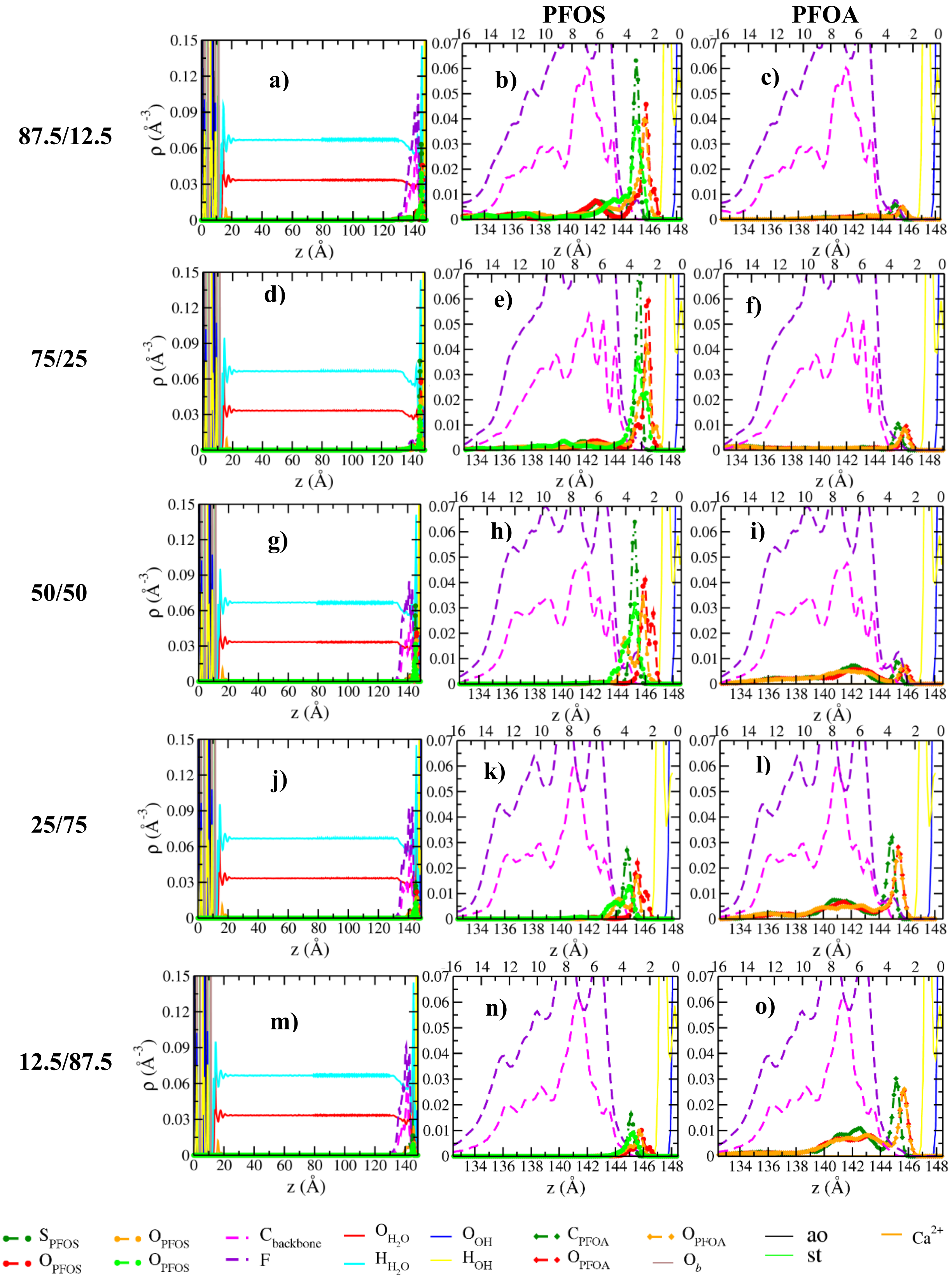
PFOS
PFOA
87.5/12.5
75/25
50/50
25/75
12.5/87.5
a)
b)
c)
d)
e)
f)
g)
h)
i)
j)
k)
l)
m)
n)
o)
ρ (Å^{-3})
z (Å)
S_{PFOS}
O_{PFOS}
O_{PFOS}
O_{PFOS}
$C_{backbone}$
F
O_{H_2O}
H_{H_2O}
O_{OH}
H_{OH}
C_{PFOA}
O_{PFOA}
O_{PFOA}
O_b
ao
st
Ca^{2+}

63

Figure S4: Computed ADP's of PFOS and PFOA molecules in Ca-kaolinite as functions of distance normal to the basal hydroxyl surface for three different PFOS-PFOA binary mixture compositions. a) - c) 87.5/12.5 (%); d) - f) – 75/25 (%); g) - i) 50/50 (%); j) - l) – 25/75 (%); m) - o) – 12.5/87.5 (%). Left column represents the whole simulation box while the middle and right columns represent PFOS and PFOA, respectively. The '*z*' values in the bottom x-axis represent the actual positions of the PFAS and H_2O molecules in the simulation box (see Figure S1a). The '*z*' values in the top x-axis of the middle and right column plots corresponds to the distances of PFAS and H_2O molecules from the plane corresponding to the peak maxima of the basal surface hydroxyl oxygen (O_{OH}) atoms of kaolinite which is set as the origin on the right side (0.0 Å).

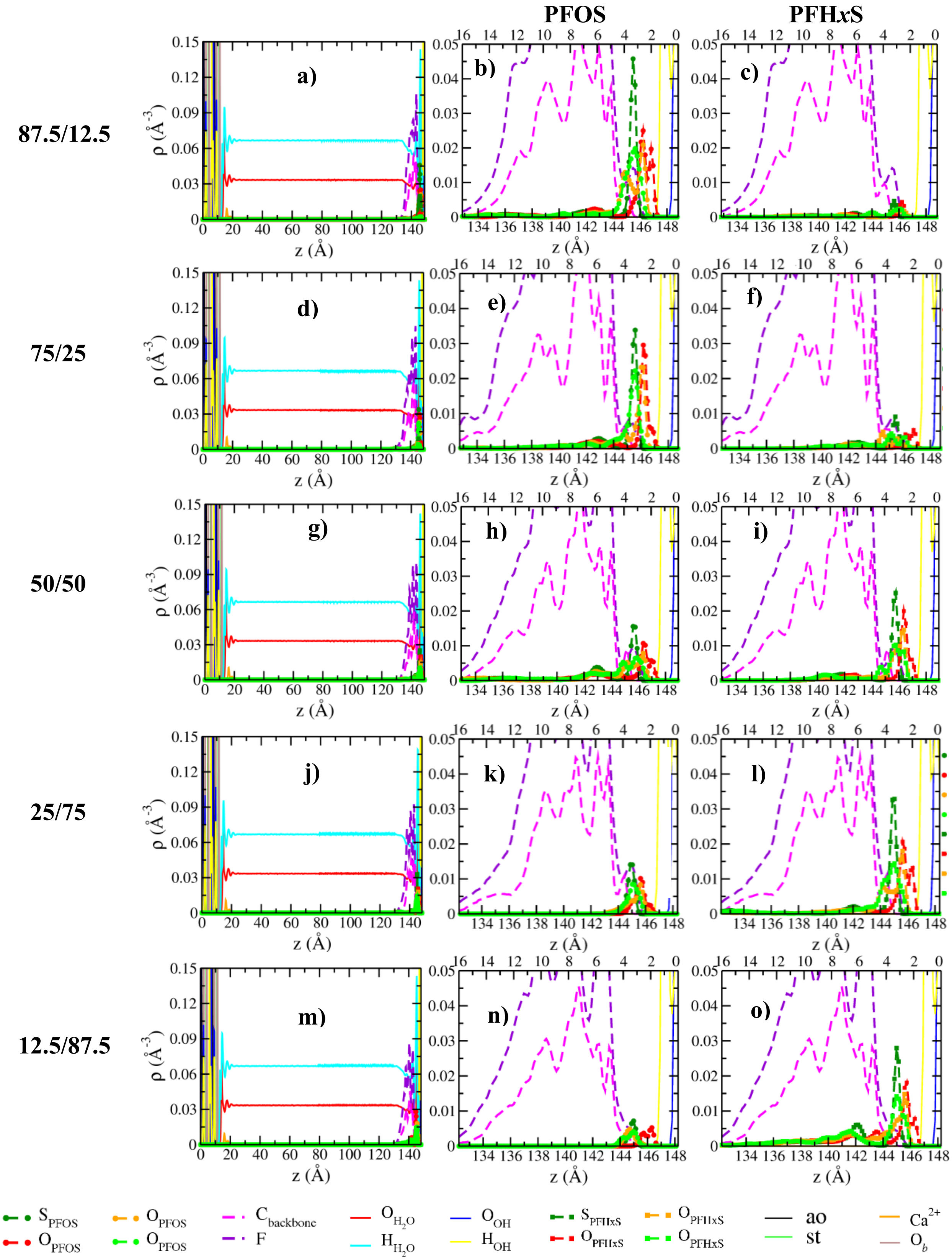
PFOS
PFHxS
87.5/12.5
75/25
50/50
25/75
12.5/87.5
a)
b)
c)
d)
e)
f)
g)
h)
i)
j)
k)
l)
m)
n)
o)
ρ (Å⁻³)
z (Å)
0 20 40 60 80 100 120 140
134 136 138 140 142 144 146 148
16 14 12 10 8 6 4 2 0
0 0.03 0.06 0.09 0.12 0.15
0 0.01 0.02 0.03 0.04 0.05
S_PFOS
O_PFOS
O_PFOS
O_PFOS
C_backbone
F
O_H2O
H_H2O
O_OH
H_OH
S_PFHxS
O_PFHxS
O_PFHxS
O_PFHxS
ao
st
Ca2+
O_b

75

Figure S5: Computed ADP's of PFOS and PFH*x*S molecules in Ca-kaolinite as functions of distance normal to the basal hydroxyl surface for three different PFOS-PFH*x*S binary mixture compositions. a) - c) 87.5/12.5 (%); d) - f) – 75/25 (%); g) - i) 50/50 (%); j) - l) – 25/75 (%); m) - o) – 12.5/87.5 (%). Left column represents the whole simulation box while the middle and right columns represent PFOS and PFH*x*S, respectively. The '*z*' values in the bottom x-axis represent the actual positions of the PFAS and H_2O molecules in the simulation box (see Figure S1a). The '*z*' values in the top x-axis of the middle and right column plots corresponds to the distances of PFAS and H_2O molecules from the plane corresponding to the peak maxima of the basal surface hydroxyl oxygen (O_{OH}) atoms of kaolinite which is set as the origin on the right side (0.0 Å).

ADP's of H_2O and Ca^{2+} ions

The ADP's of H_2O molecules as functions of distance normal to the basal hydroxyl surface of Ca-kaolinite demonstrates that the interfacial H_2O adsorption characteristics are not significantly influenced between two different types of binary mixture composition and their concentrations (Figure 1a-1c and 2a-2c). For instance, the O_{H2O} exhibits two dominant peaks at distances ~2.8 and ~6.3 Å away from the basal hydroxyl surface oxygen atoms along with broad peaks at larger distances. The well-defined peaks at ~2.8 Å and the presence of H_{H2O} peak at ~1.8 Å evidently indicates that the surface adsorbed water molecules exhibit direct H-bonding interaction with the basal surface 'H' atoms of the hydroxyl groups. Importantly, the first ADP peaks of O_{H2O} and H_{H2O} at the siloxane surface show broad distributions (in comparison to hydroxyl surface) which should primarily be attributed to the hydrophobic character (Figure S6). The reported ADP's are consistent with previous simulations on different PFAS and other organics. [1–4] In addition, due to the hydrophobic nature of the surface, the Ca^{2+} ions do not exhibit coordination with the basal surface which is evident by their ADP peaks at ~4.6 Å and a broad shoulder between 5.8-9.0 Å away from the basal siloxane surface as illustrated in Figure S6.

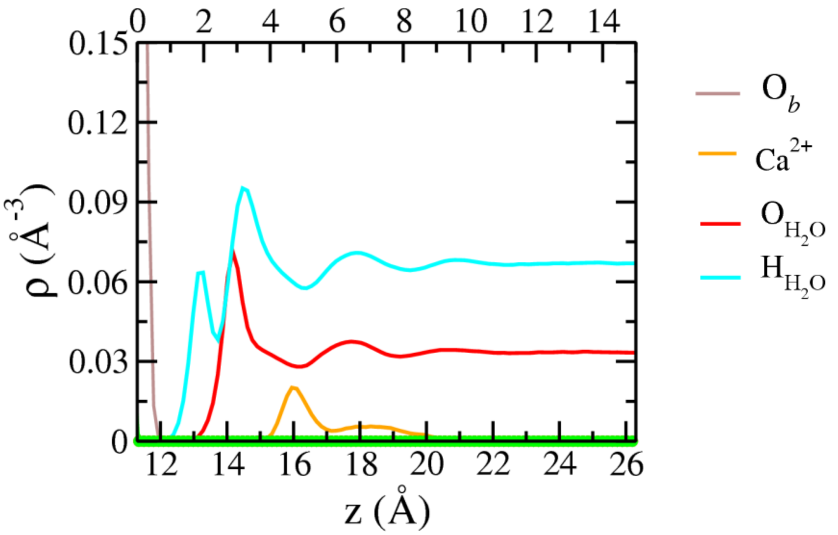

Figure S6: Computed ADP's of Ca^{2+} ions and H_2O molecules in Ca-kaolinite as functions of distance normal to the basal oxygen atoms (O_b) of siloxane surface for binary mixture. The '*z*' values in the bottom x-axis represent the actual positions of the Ca^{2+} ions and H_2O molecules in the simulation box (see Figure S1a). The '*z*' values in the top x-axis corresponds to the distances of Ca^{2+} and H_2O molecules from the plane corresponding to the peak maxima of the basal surface oxygen (O_b) atoms of siloxane surface of kaolinite which is set as the origin (0.0 Å).

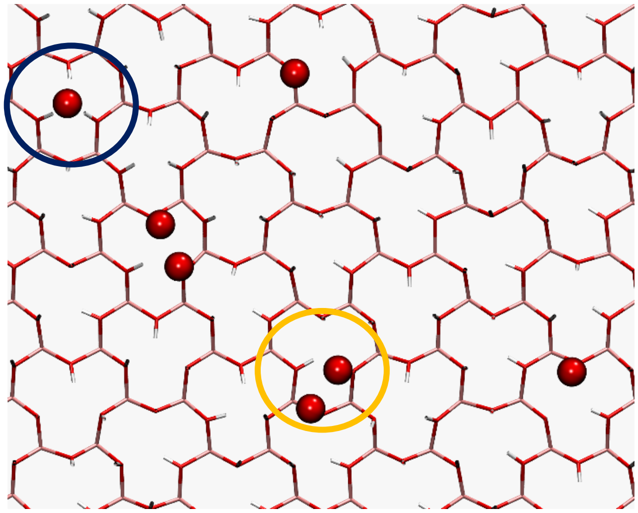

Figure S7: Pictorial representations of surface adsorbed 'O' atoms of SO_3 groups in PFOS at the basal hydroxyl surface of Ca-kaolinite. Blue circle represents the PFOS molecules with the 'O' atoms of SO_3 groups at the center of the hydroxyl cavity at z – 2.0 Å; Orange circle represents the PFOS molecules with the 'O' atoms of SO_3 groups located directly on top of the hydroxyl groups resulting in an ADP peak at z – 2.7 Å.

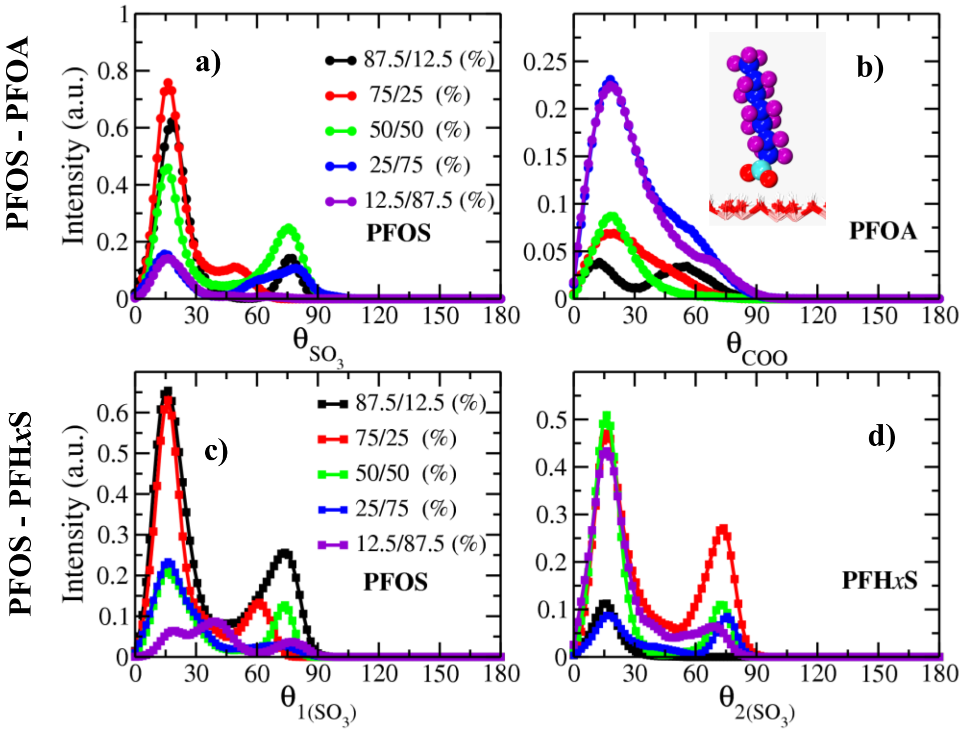

Figure S8: Computed orientation distributions of only surface adsorbed PFOS, PFOA and PFH*x*S molecules with respect to surface normal of Ca-kaolinite at all binary mixture concentrations. a) and b) PFOS-PFOA; c) and d) PFOS-PFH*x*S. θ_{SO3} and θ_{COO} represent the angle between the vector bisecting the SO_3/COO groups of PFOS – PFOA binary mixture. $\theta_{1(SO3)}$ and $\theta_{2(SO3)}$ represent the angle between the vector bisecting the SO_3 groups of PFOS – PFH*x*S binary mixtures.

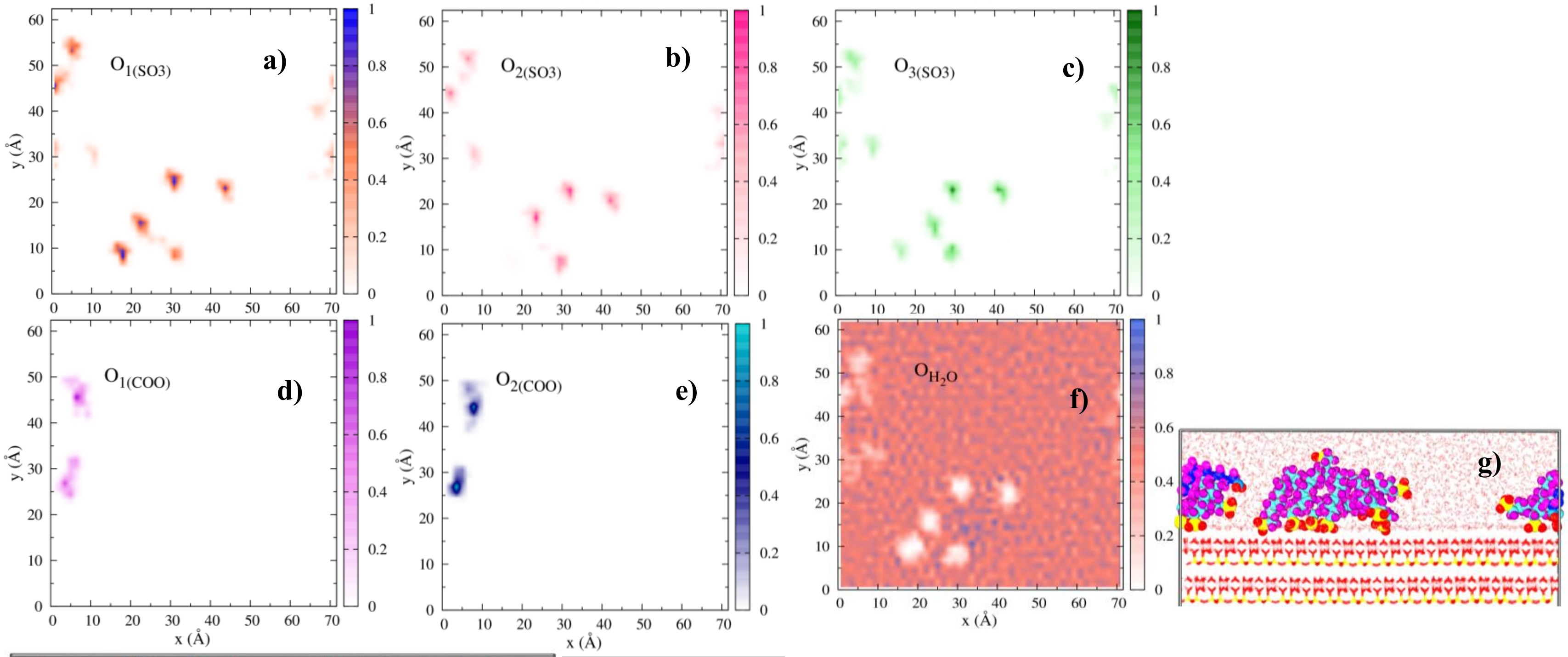

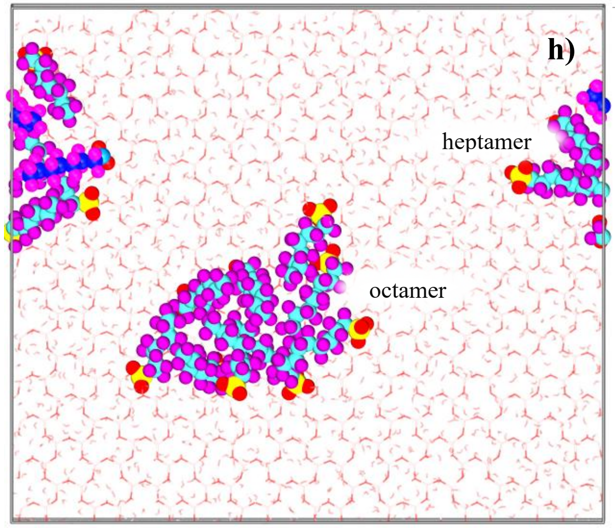

Figure S9: Computed PDM's of surface adsorbed PFOS, PFOA and H_2O molecules in Ca-kaolinite on the basal hydroxyl surface for 87.5/12.5 (%) PFOS-PFOA binary mixture composition. a) $O_{1(SO3)}$, b) $O_{2(SO3)}$ and c) $O_{3(SO3)}$ - three 'O' atoms of SO_3 groups in PFOS; d) $O_{1(COO)}$ and e) $O_{2(COO)}$ – two 'O' atoms of COO groups in PFOA; f) 'O' aotms of H_2O. g) and h) Pictorial representation of PFOS and PFOA at the interfacial region of external (001) hydroxyl surface of Ca-kaolinite. Color codes: pink sticks – Al octahedra; red sticks – hydroxyl 'O' atoms; white sticks – hydroxyl 'H' atoms; cyan spheres – carbon backbone of PFOS; purple spheres – F; yellow spheres – 'S' atom of SO_3 groups; blue spheres – carbon back bone of PFOA; red spheres – 'O' atoms of SO_3 groups. H_2O molecules are shown in transparent representation for clarity.

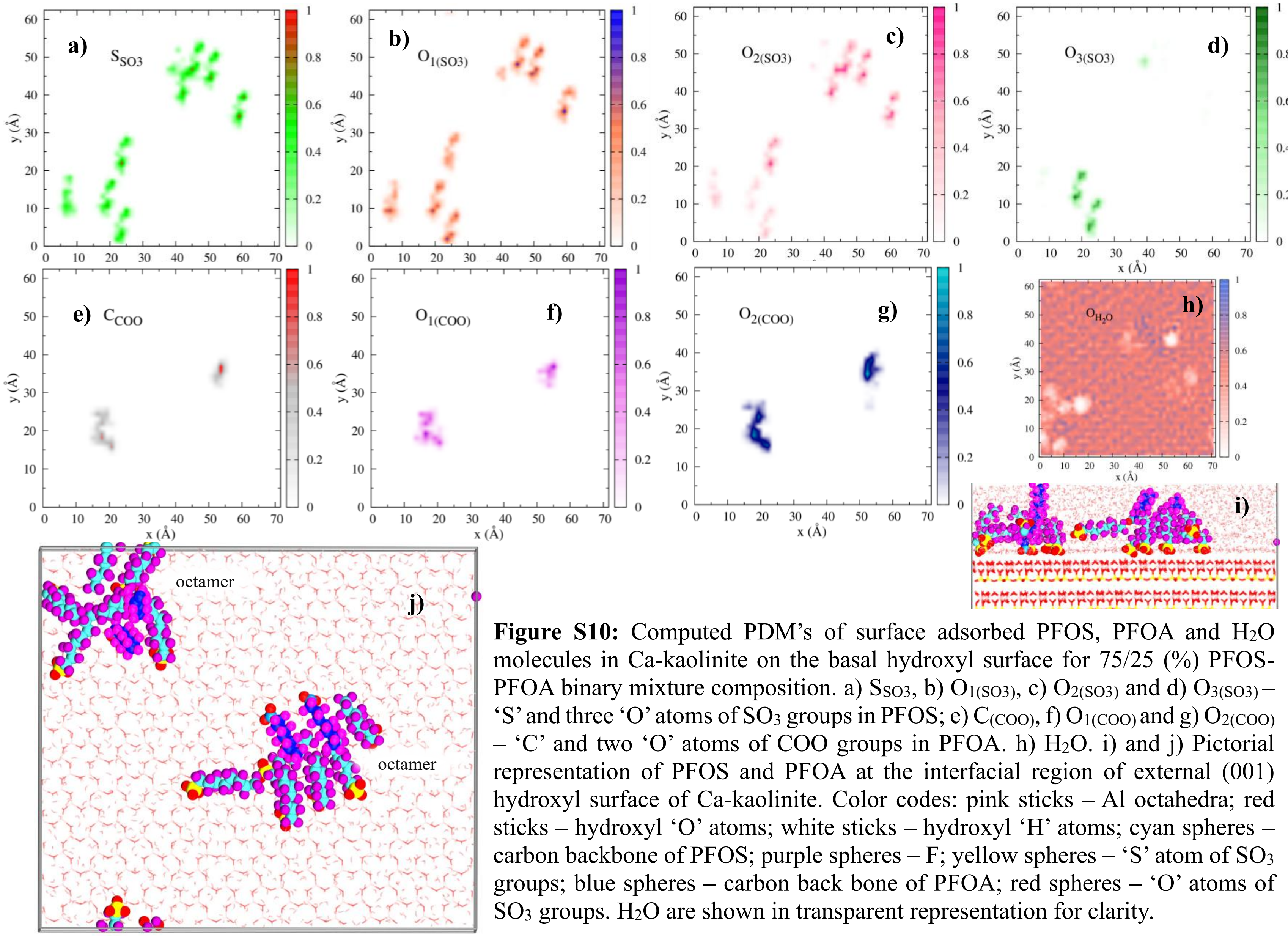

Figure S10: Computed PDM's of surface adsorbed PFOS, PFOA and H_2O molecules in Ca-kaolinite on the basal hydroxyl surface for 75/25 (%) PFOS-PFOA binary mixture composition. a) S_{SO3}, b) $O_{1(SO3)}$, c) $O_{2(SO3)}$ and d) $O_{3(SO3)}$ – 'S' and three 'O' atoms of SO_3 groups in PFOS; e) $C_{(COO)}$, f) $O_{1(COO)}$ and g) $O_{2(COO)}$ – 'C' and two 'O' atoms of COO groups in PFOA. h) H_2O. i) and j) Pictorial representation of PFOS and PFOA at the interfacial region of external (001) hydroxyl surface of Ca-kaolinite. Color codes: pink sticks – Al octahedra; red sticks – hydroxyl 'O' atoms; white sticks – hydroxyl 'H' atoms; cyan spheres – carbon backbone of PFOS; purple spheres – F; yellow spheres – 'S' atom of SO_3 groups; blue spheres – carbon back bone of PFOA; red spheres – 'O' atoms of SO_3 groups. H_2O are shown in transparent representation for clarity.

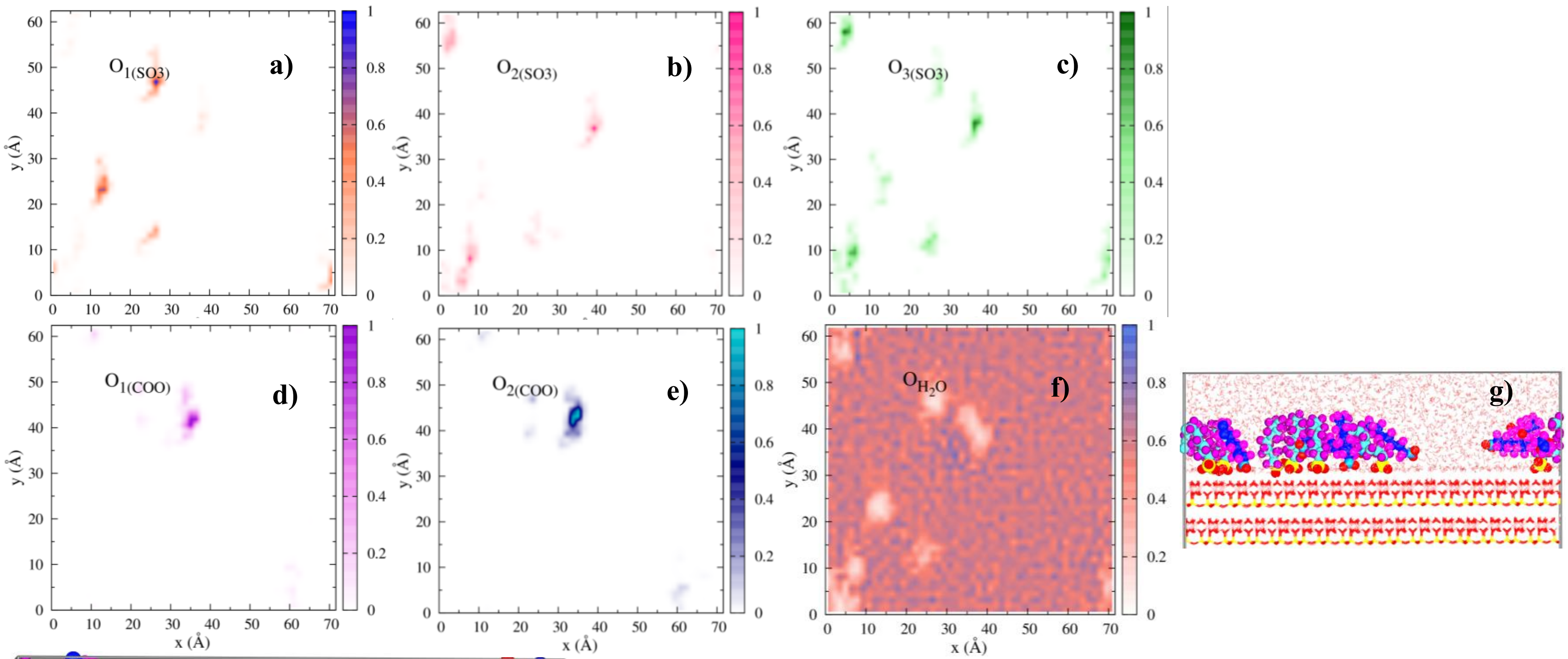

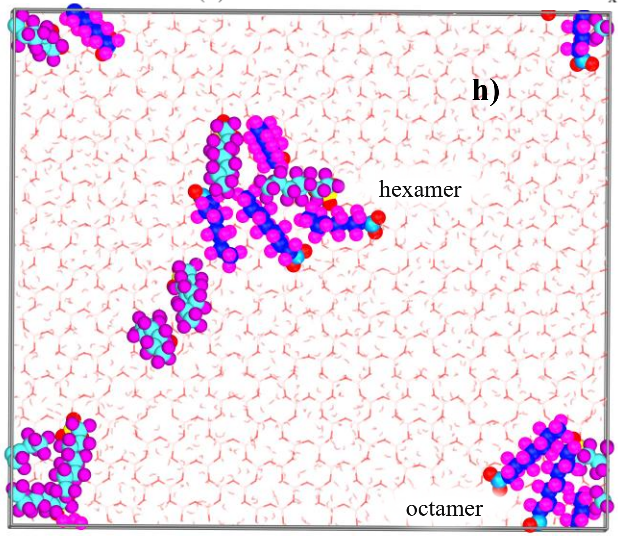

Figure S11: Computed PDM's of surface adsorbed PFOS, PFOA and H_2O molecules in Ca-kaolinite on the basal hydroxyl surface for 50/50 (%) PFOS-PFOA binary mixture composition. a) $O_{1(SO3)}$, b) $O_{2(SO3)}$ and c) $O_{3(SO3)}$ - three 'O' atoms of SO_3 groups in PFOS; d) $O_{1(COO)}$ and e) $O_{2(COO)}$ – two 'O' atoms of COO groups in PFOA. ; f) 'O' aotms of H_2O. g) and h) Pictorial representation of PFOS and PFOA at the interfacial region of external (001) hydroxyl surface of Ca-kaolinite. Color codes: pink sticks – Al octahedra; red sticks – hydroxyl 'O' atoms; white sticks – hydroxyl 'H' atoms; cyan spheres – carbon backbone of PFOS; purple spheres – F; yellow spheres – 'S' atom of SO_3 groups; blue spheres – carbon back bone of PFOA; red spheres – 'O' atoms of SO_3 groups. H_2O molecules are shown in transparent representation for clarity.

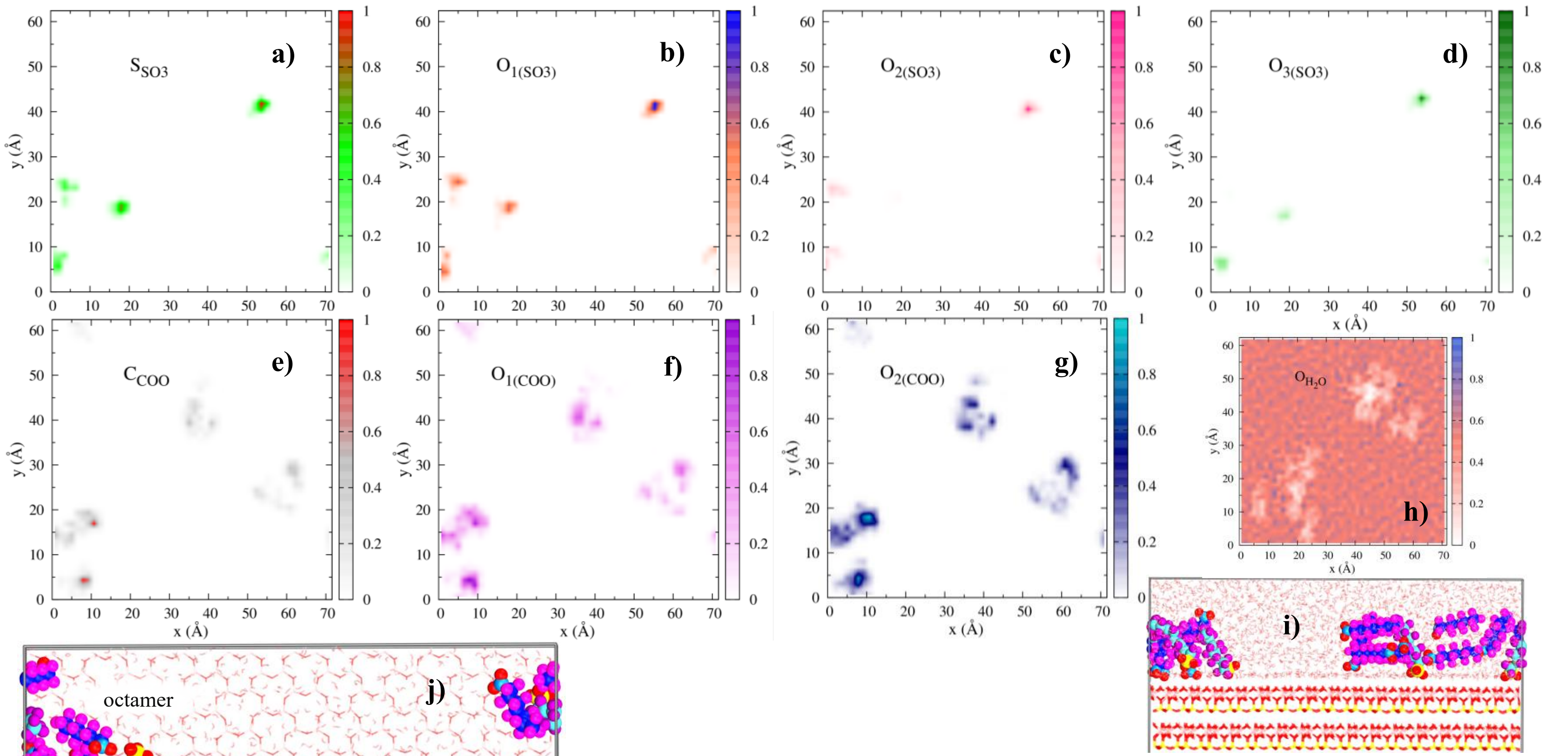

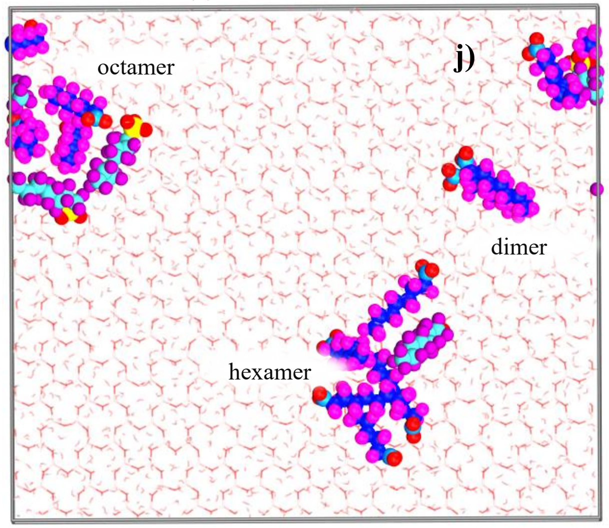

Figure S12: Computed PDM's of surface adsorbed PFOS, PFOA and H_2O molecules in Ca-kaolinite on the basal hydroxyl surface for 25/75 (%) PFOS-PFOA binary mixture composition. a) S_{SO3}, b) $O_{1(SO3)}$, c) $O_{2(SO3)}$ and d) $O_{3(SO3)}$ – 'S' and three 'O' atoms of SO_3 groups in PFOS; e) $C_{(COO)}$, f) $O_{1(COO)}$ and g) $O_{2(COO)}$ – 'C' and two 'O' atoms of COO groups in PFOA. h) H_2O. i) and j) Pictorial representation of PFOS and PFOA at the interfacial region of external (001) hydroxyl surface of Ca-kaolinite. Color codes: pink sticks – Al octahedra; red sticks – hydroxyl 'O' atoms; white sticks – hydroxyl 'H' atoms; cyan spheres – carbon backbone of PFOS; purple spheres – F; yellow spheres – 'S' atom of SO_3 groups; blue spheres – carbon back bone of PFOA; red spheres – 'O' atoms of SO_3 groups. H_2O molecules are shown in transparent representation for clarity.

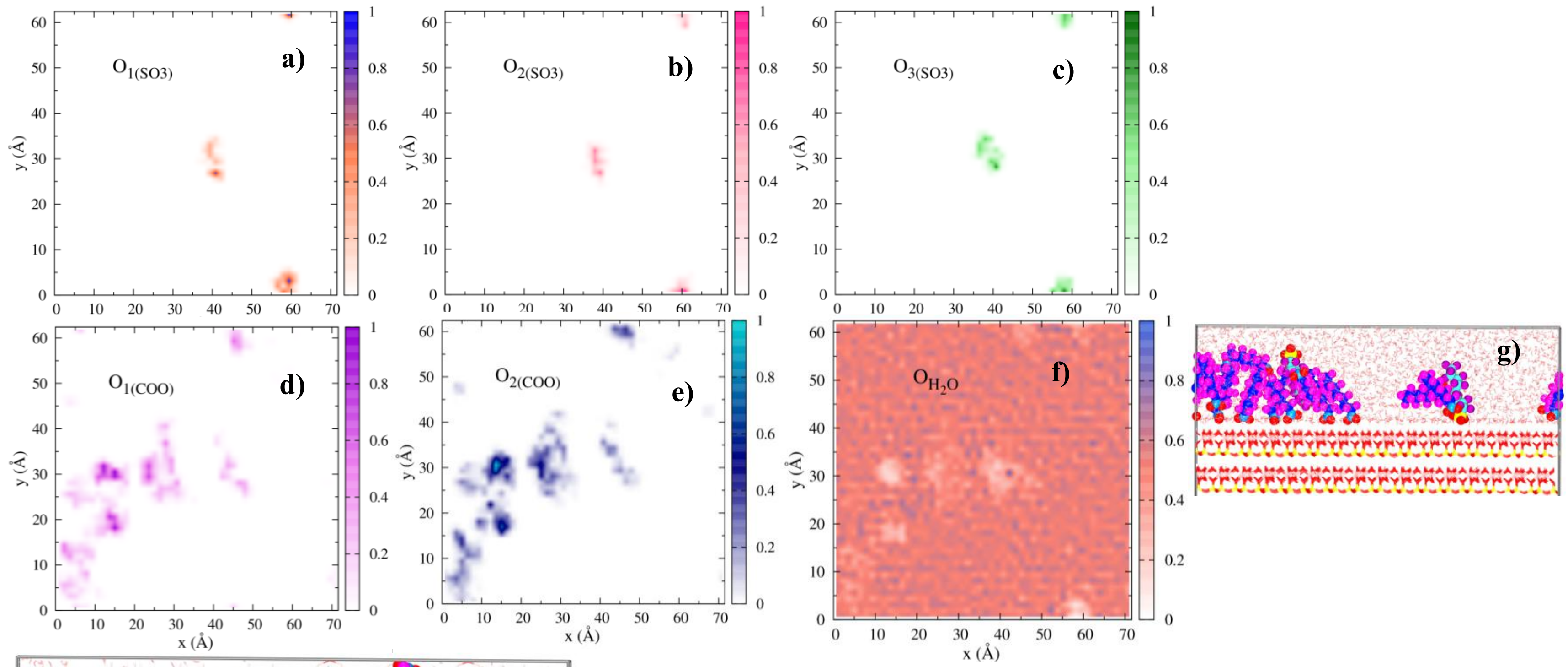

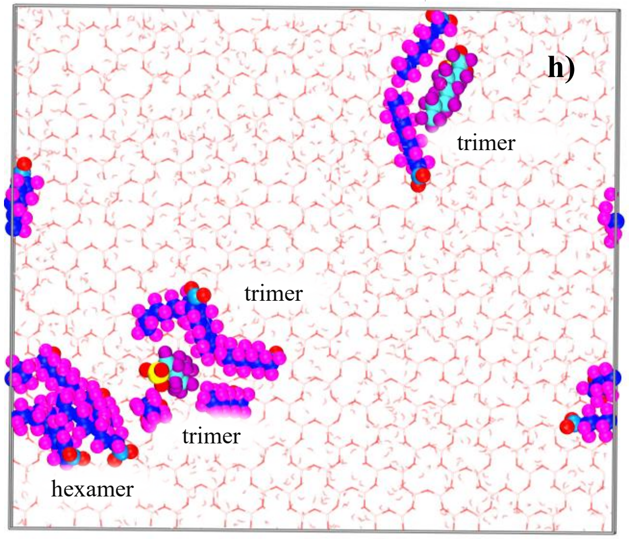

Figure S13: Computed PDM's of surface adsorbed PFOS, PFOA and H_2O molecules in Ca-kaolinite on the basal hydroxyl surface for 12.5/87.5 (%) PFOS-PFOA binary mixture composition. a) $O_{1(SO3)}$, b) $O_{2(SO3)}$ and c) $O_{3(SO3)}$ - three 'O' atoms of SO_3 groups in PFOS; d) $O_{1(COO)}$ and e) $O_{2(COO)}$ – two 'O' atoms of COO groups in PFOA; f) 'O' aotms of H_2O. g) and h) Pictorial representation of PFOS and PFOA at the interfacial region of external (001) hydroxyl surface of Ca-kaolinite. Color codes: pink sticks – Al octahedra; red sticks – hydroxyl 'O' atoms; white sticks – hydroxyl 'H' atoms; cyan spheres – carbon backbone of PFOS; purple spheres – F; yellow spheres – 'S' atom of SO_3 groups; blue spheres – carbon back bone of PFOA; red spheres – 'O' atoms of SO_3 groups. H_2O molecules are shown in transparent representation for clarity.

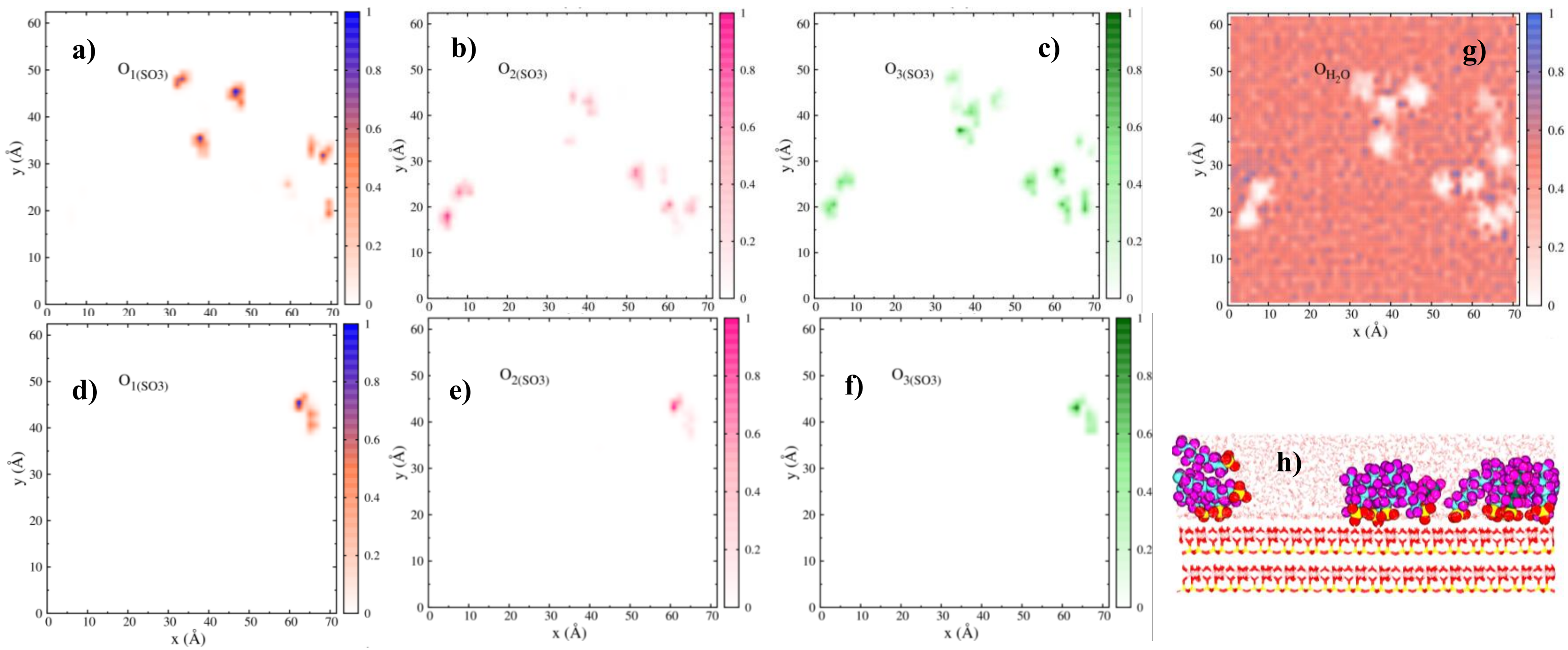

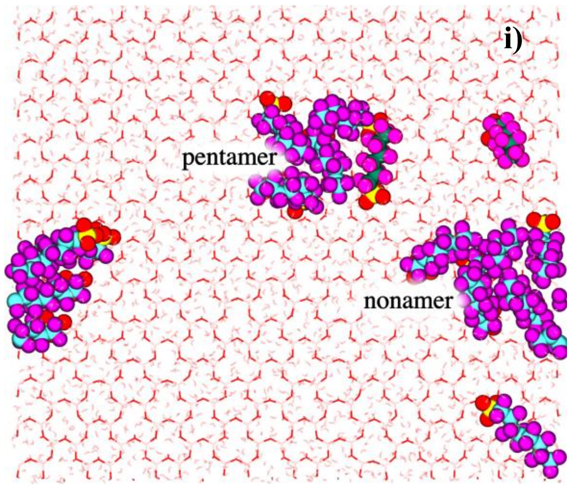

Figure S14: Computed PDM's of surface adsorbed PFOS, PFH*x*S and H_2O molecules in Ca-kaolinite on the basal hydroxyl surface for 87.5/12.5 (%) PFOS-PFH*x*S binary mixture composition. $O_{1(SO3)}$, $O_{2(SO3)}$ and $O_{3(SO3ss)}$ – three 'O' atoms of SO_3 groups in PFOS (a-c) and PFH*x*S (d-f); g) 'O' aotms of H_2O. h) and i) Pictorial representation of PFOS and PFH*x*S at the interfacial region of external (001) hydroxyl surface of Ca-kaolinite. Color codes: pink sticks – Al octahedra; red sticks – hydroxyl 'O' atoms; white sticks – hydroxyl 'H' atoms; cyan spheres – carbon backbone of PFOS; purple spheres – F; yellow spheres – 'S' atom of SO_3 groups of PFOS; green spheres – carbon back bone of PFH*x*S; red spheres – 'O' atoms of SO_3 groups of PFH*x*S. H_2O molecules are shown in transparent representation for clarity.

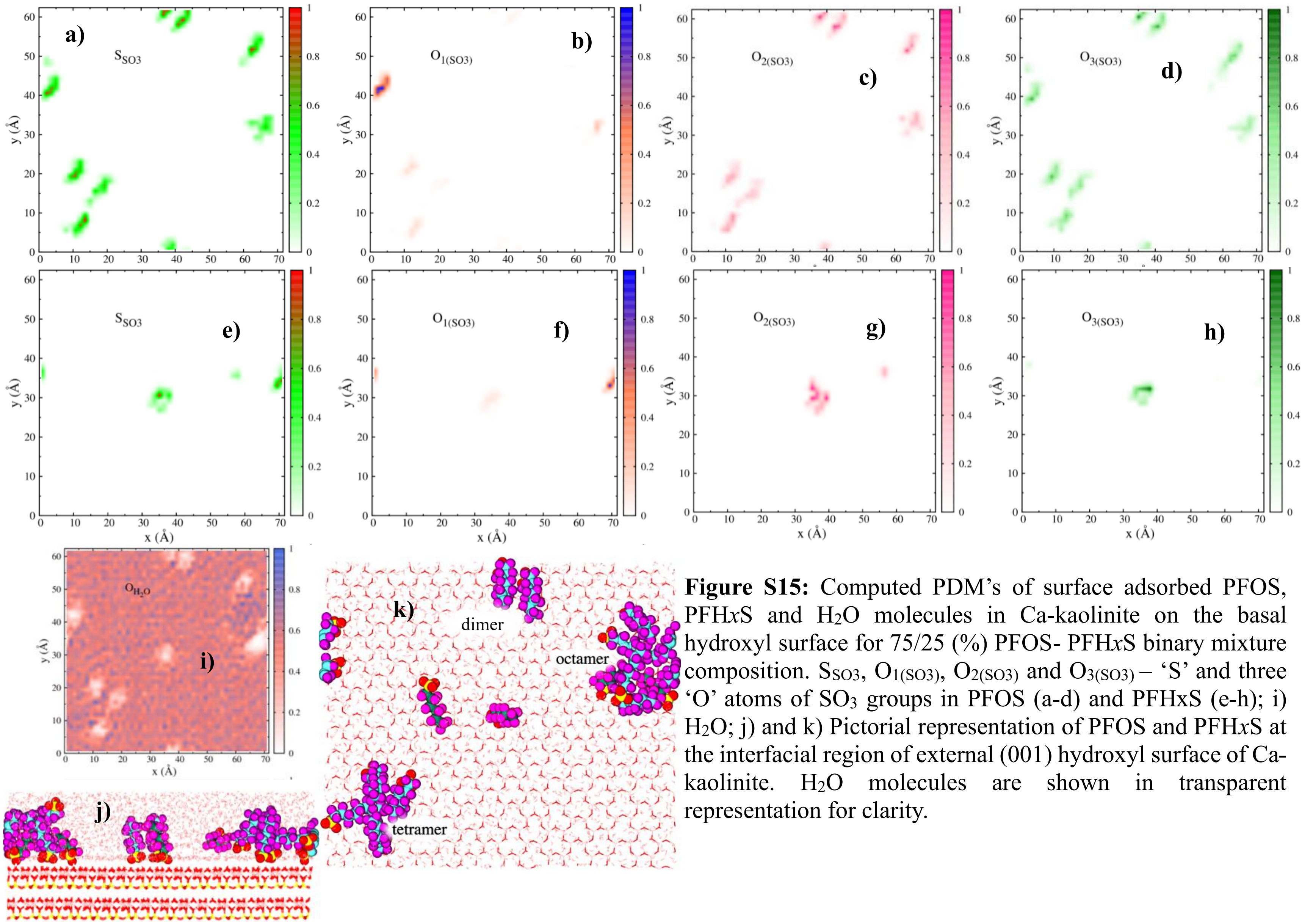

Figure S15: Computed PDM's of surface adsorbed PFOS, PFH*x*S and H_2O molecules in Ca-kaolinite on the basal hydroxyl surface for 75/25 (%) PFOS- PFH*x*S binary mixture composition. S_{SO3}, $O_{1(SO3)}$, $O_{2(SO3)}$ and $O_{3(SO3)}$ – 'S' and three 'O' atoms of SO_3 groups in PFOS (a-d) and PFHxS (e-h); i) H_2O; j) and k) Pictorial representation of PFOS and PFH*x*S at the interfacial region of external (001) hydroxyl surface of Ca-kaolinite. H_2O molecules are shown in transparent representation for clarity.

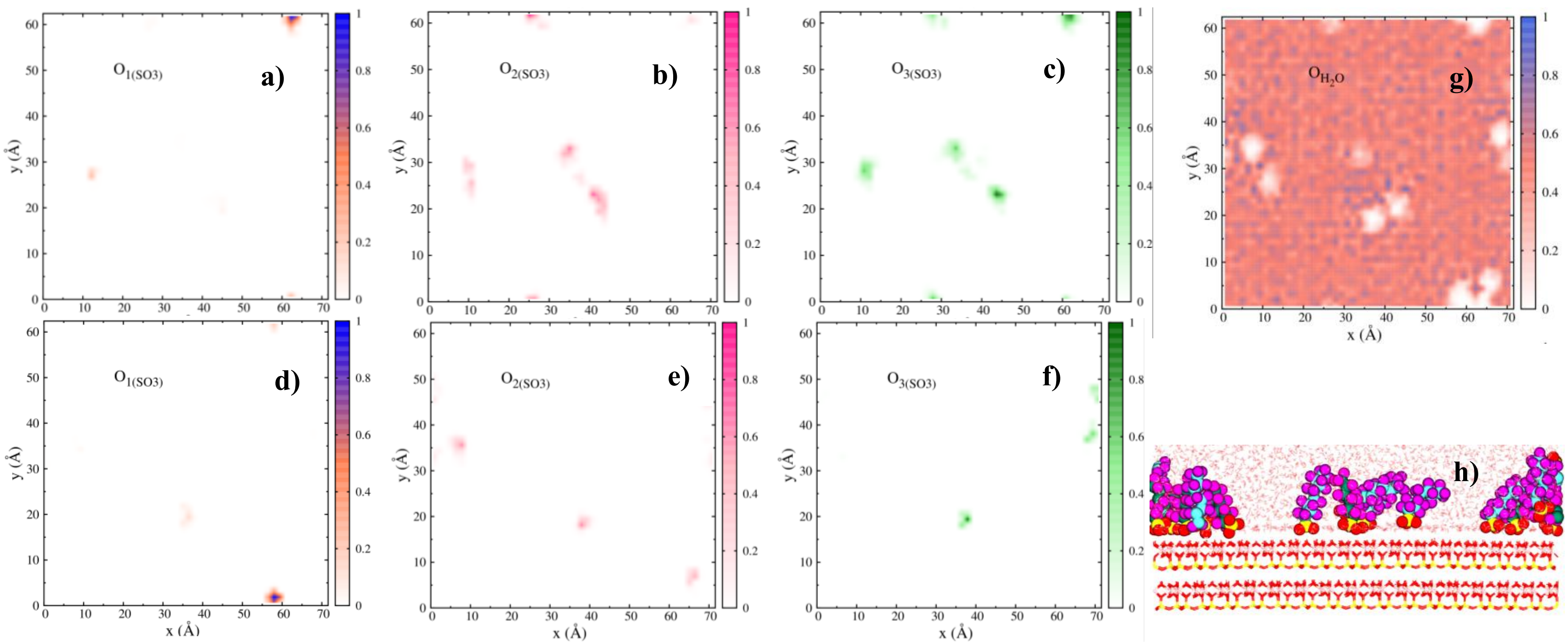

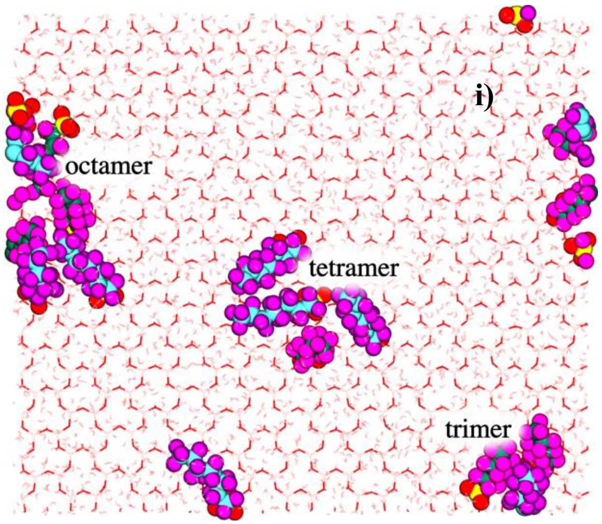

Figure S16: Computed PDM's of surface adsorbed PFOS, PFH*x*S and H_2O molecules in Ca-kaolinite on the basal hydroxyl surface for 50/50 (%) PFOS-PFH*x*S binary mixture composition. $O_{1(SO3)}$, $O_{2(SO3)}$ and $O_{3(SO3)}$ – three 'O' atoms of SO_3 groups in PFOS (a-c) and PFH*x*S (d-f); g) 'O' aotms of H_2O. h) and i) Pictorial representation of PFOS and PFH*x*S at the interfacial region of external (001) hydroxyl surface of Ca-kaolinite. Color codes: pink sticks – Al octahedra; red sticks – hydroxyl 'O' atoms; white sticks – hydroxyl 'H' atoms; cyan spheres – carbon backbone of PFOS; purple spheres – F; yellow spheres – 'S' atom of SO_3 groups of PFOS; green spheres – carbon back bone of PFH*x*S; red spheres – 'O' atoms of SO_3 groups of PFH*x*S. H_2O molecules are shown in transparent representation for clarity.

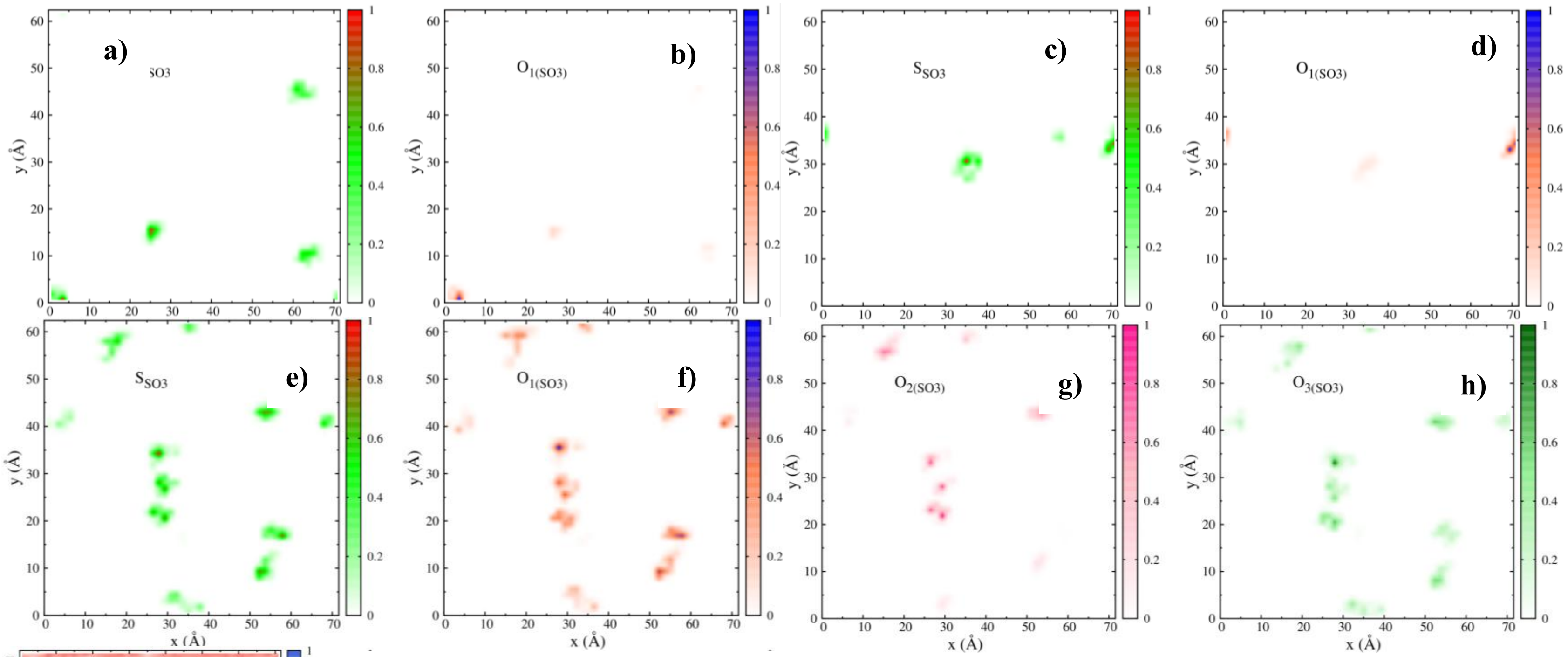

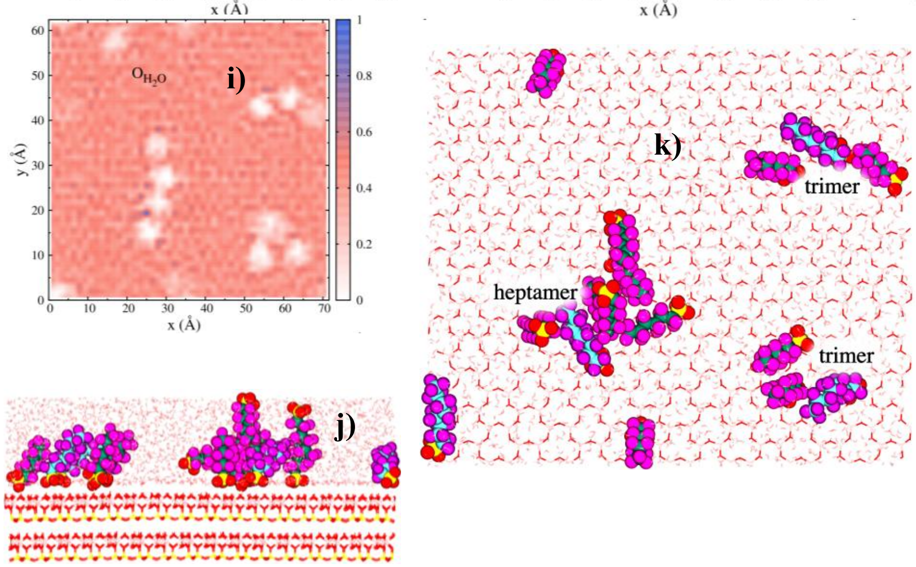

Figure S17: Computed PDM's of surface adsorbed PFOS, PFH*x*S and H_2O molecules in Ca-kaolinite on the basal hydroxyl surface for 25/75 (%) PFOS- PFH*x*S binary mixture composition. S_{SO3}, $O_{1(SO3)}$, $O_{2(SO3)}$ and $O_{3(SO3)}$ – 'S' and three 'O' atoms of SO_3 groups in PFOS (a-d) and PFH*x*S (e-h); i) H_2O; j) and k) Pictorial representation of PFOS and PFH*x*S at the interfacial region of external (001) hydroxyl surface of Ca-kaolinite. Color codes: pink sticks – Al octahedra; red sticks – hydroxyl 'O' atoms; white sticks – hydroxyl 'H' atoms; cyan spheres – carbon backbone of PFOS; purple spheres – F; yellow spheres – 'S' atom of SO_3 groups of PFOS; green spheres – carbon back bone of PFH*x*S; red spheres – 'O' atoms of SO_3 groups of PFH*x*S.

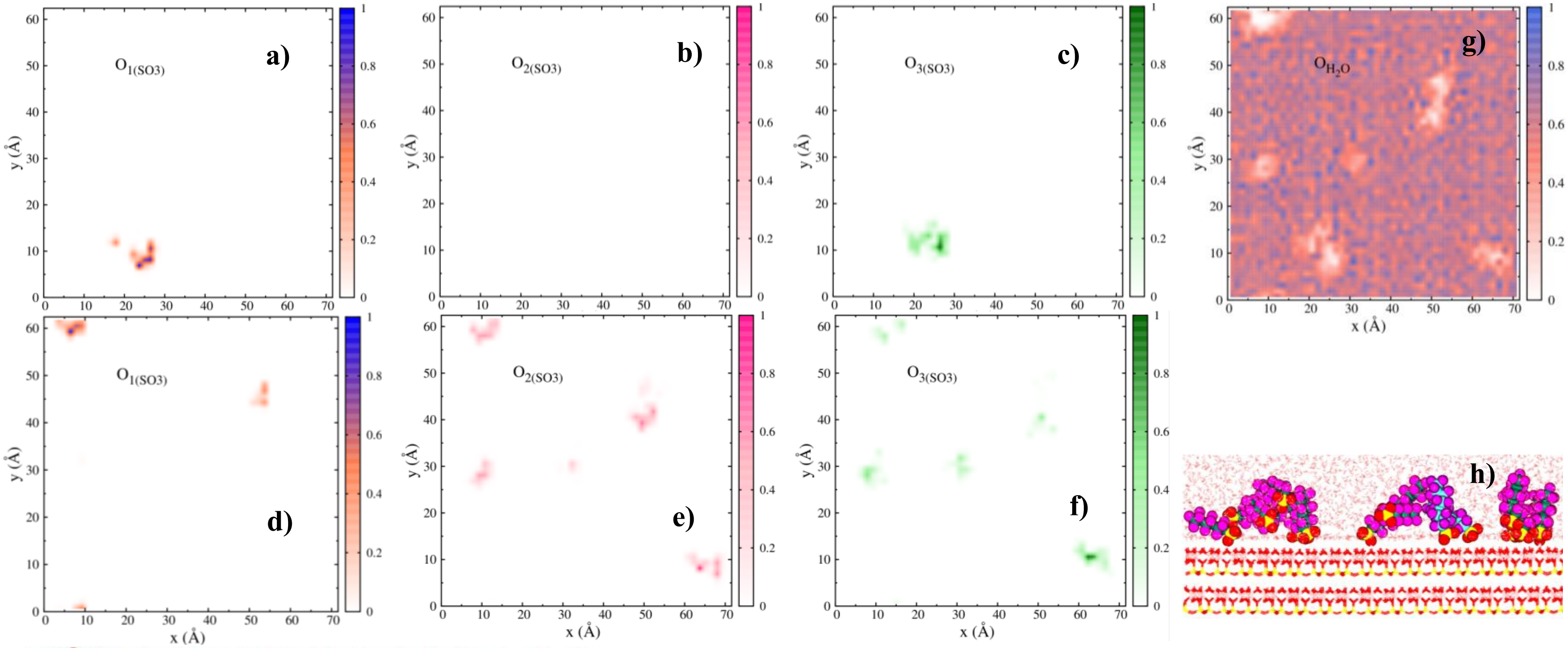

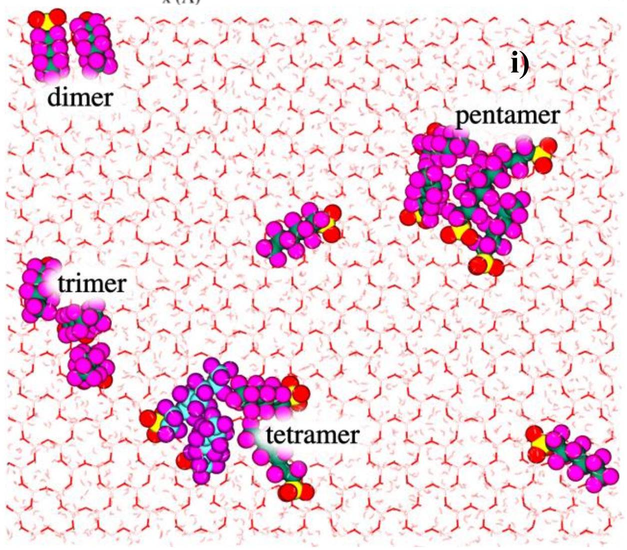

Figure S18: Computed PDM's of surface adsorbed PFOS, PFH*x*S and H_2O molecules in Ca-kaolinite on the basal hydroxyl surface for 12.5/87.5 (%) PFOS- PFH*x*S binary mixture composition. $O_{1(SO3)}$, $O_{2(SO3)}$ and $O_{3(SO3)}$ – three 'O' atoms of SO_3 groups in PFOS (a-c) and PFH*x*S (d-f); g) 'O' aotms of H_2O. h) and i) Pictorial representation of PFOS and PFH*x*S at the interfacial region of external (001) hydroxyl surface of Ca-kaolinite. sColor codes: pink sticks – Al octahedra; red sticks – hydroxyl 'O' atoms; white sticks – hydroxyl 'H' atoms; cyan spheres – carbon backbone of PFOS; purple spheres – F; yellow spheres – 'S' atom of SO_3 groups of PFOS; green spheres – carbon back bone of PFH*x*S; red spheres – 'O' atoms of SO_3 groups of PFH*x*S. H_2O molecules are shown in transparent representation for clarity.

Coordination Details

The interaction of PFAS molecules with the basal surface hydroxyl atoms were examined for both types of binary mixtures. From Figure S19a and S19b, irrespective of the concentrations of PFOS/PFOA binary mixtures, the adsorption of PFOS and PFOA is characterized by the interaction of terminal functional groups with the 'H' atoms of the surface hydroxyl groups. However, the running coordination number (RCN) of PFOS varies slightly (~0.5 - 0.7) with a decrease in their concentration in the binary mixture. Such marginal variations in the RCN could be attributed to the distributions of surface adsorbed PFOS molecules in two adsorption sites as shown in Figure S7. At the same time, regardless of the concentration of PFOA in the PFOS/PFOA binary mixture, the RCN values are ~0.5 which show that both 'O' atoms of the surface adsorbed COO^- group exhibit H-bond interaction with the surface hydroxyl 'H' atoms. The reported RCN's are in good agreement with previous studies of single-phase PFOA interactions with clays.[37,38]

Similarly, Figure 19c and 19d illustrate that the adsorption of PFOS and PFH*x*S molecules are primarily due to the interaction between the terminal functional group atoms and the surface hydroxyl 'H' atoms. Notably, the RCN of PFOS is similar despite the decrease in their concentration in the binary mixture (<50%). For instance, the RCN of PFOS with surface atoms are on average ~0.5-0.6 for all binary mixture concentrations examined, except for the systems with higher percentages of PFH*x*S (12.5/87.5 %). The higher RCN values for these high PFH*x*S concentration systems results from the interaction of all PFOS molecules with the basal surface atoms as shown in Figures S18a and 4e. On the other hand, the RCN of PFH*x*S is ~0.5 irrespective of their concentration in the binary mixture. This suggests that PFOS is preferentially adsorbed to the surface over PFH*x*S even at low concentrations.

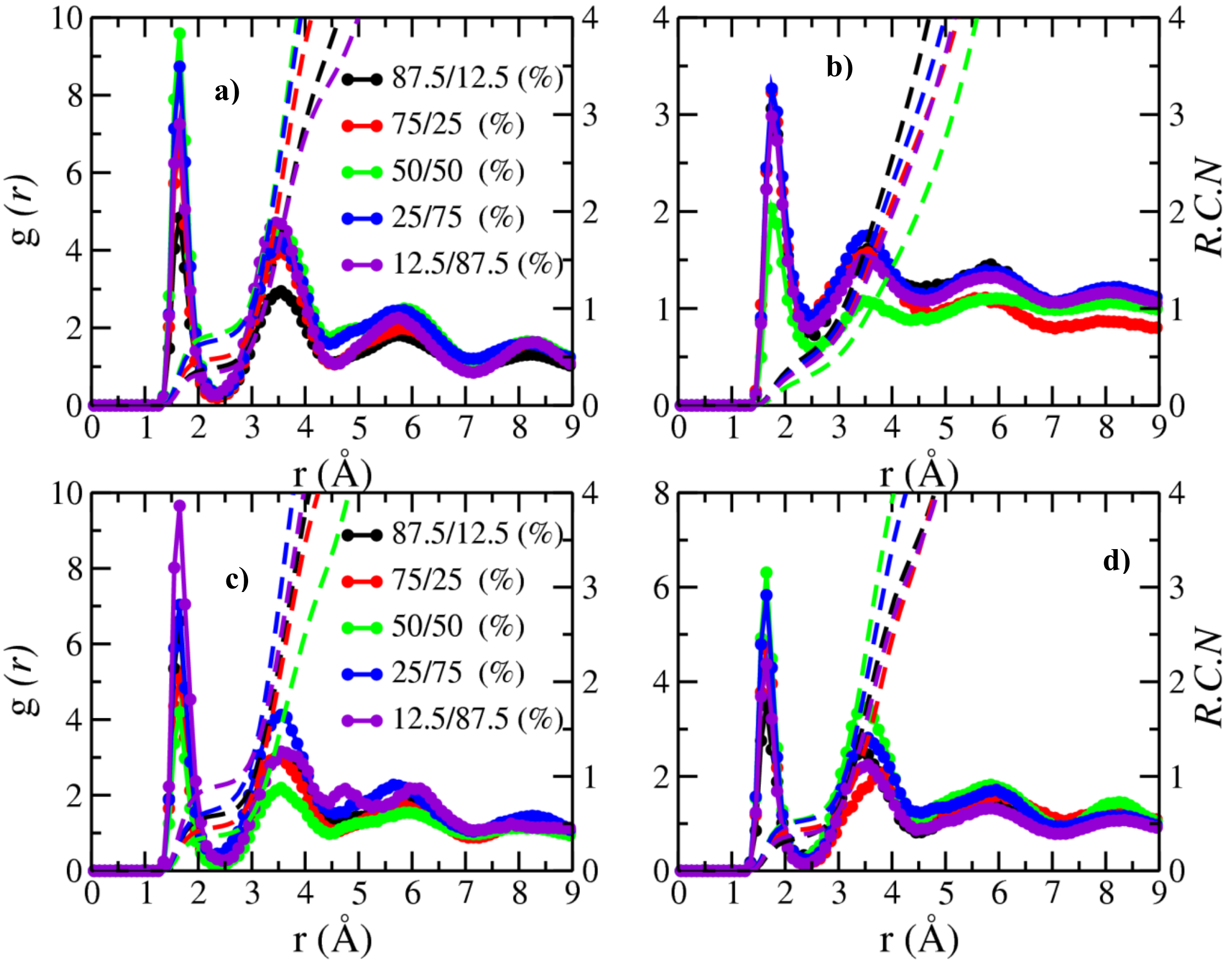

Figure S19: Radial distribution functions (solid lines) and the corresponding running coordination numbers (dashed lines) between PFAS and the 'H' atoms of the basal surface hydroxyl groups for different binary mixture compositions of PFOS/PFOA (a and b) and PFOS/PFHxS (c and d) in Ca-kaolinite. a) O_{SO3} (PFOS) - 'H' atoms of hydroxyl groups; b) O_{COO} (PFOA) - 'H' atoms of hydroxyl groups; c) O_{SO3} (PFOS) - 'H' atoms of hydroxyl groups and d) O_{SO3} (PFH*x*S) - 'H' atoms of hydroxyl groups.

Concentration (%)	Mixture Composition	L_x (Å)	L_y (Å)	L_z (Å)
**87.5 / 12.5**	PFOS - PFOA	71.55	62.45	148.38
	PFOS - PFH*x*S	71.55	62.38	148.90
**75 / 25**	PFOS - PFOA	71.52	62.47	149.10
	PFOS - PFH*x*S	71.56	62.38	148.73
**50 / 50**	PFOS - PFOA	71.56	62.40	148.59
	PFOS - PFH*x*S	71.56	62.45	148.94
**25 / 75**	PFOS - PFOA	71.57	62.41	148.12
	PFOS - PFH*x*S	71.58	62.35	148.35
**12.5 / 87.5**	PFOS - PFOA	71.62	62.42	148.45
	PFOS - PFH*x*S	71.61	62.38	148.23

Table S1: Cell dimensions of kaolinite used in the NVT (post-NPT) simulation runs for all binary mixtures at different concentrations. The reported cell dimensions were obtained by computing averages over 10 equal time blocks (500 ps each) during the 5 ns data production in the NPT ensemble.

Diffusion:

The diffusion coefficients of PFAS clusters and H_2O molecules were calculated from the mean square displacements of these species using the following relationship

$$\langle x^2 \rangle = 2dDt$$

where $\langle x^2 \rangle$ is the mean square displacement, *d* represents the dimensions, *D* diffusion coefficient and *t* is the time the displacements were averaged. For clusters, the diffusion is measured by computing the center of mass of individual clusters.
